# Carrot populations in France and Spain host a complex virome rich in previously uncharacterized viruses

**DOI:** 10.1371/journal.pone.0290108

**Published:** 2023-08-16

**Authors:** Deborah Schönegger, Armelle Marais, Bisola Mercy Babalola, Chantal Faure, Marie Lefebvre, Laurence Svanella-Dumas, Sára Brázdová, Thierry Candresse

**Affiliations:** 1 INRAE &, UMR 1332 Biology du Fruit et Pathologie, Univ. Bordeaux, Villenave d’Ornon Cedex, France; 2 Centro de Biotecnología y Genómica de Plantas (CBGP), Universidad Politécnica de Madrid (UPM) and E.T.S.I. Agronómica, Alimentaria y de Biosistemas, Campus de Montegancedo, Madrid, Spain; National Institute of Agricultural Technology (INTA), ARGENTINA

## Abstract

High-throughput sequencing (HTS) has proven a powerful tool to uncover the virome of cultivated and wild plants and offers the opportunity to study virus movements across the agroecological interface. The carrot model consisting of cultivated (*Daucus carota* ssp. *sativus*) and wild carrot (*Daucus carota* ssp. *carota*) populations, is particularly interesting with respect to comparisons of virus communities due to the low genetic barrier to virus flow since both population types belong to the same plant species. Using a highly purified double-stranded RNA-based HTS approach, we analyzed on a large scale the virome of 45 carrot populations including cultivated, wild and off-type carrots (carrots growing within the field and likely representing hybrids between cultivated and wild carrots) in France and six additional carrot populations from central Spain. Globally, we identified a very rich virome comprising 45 viruses of which 25 are novel or tentatively novel. Most of the identified novel viruses showed preferential associations with wild carrots, either occurring exclusively in wild populations or infecting only a small proportion of cultivated populations, indicating the role of wild carrots as reservoir of viral diversity. The carrot virome proved particularly rich in viruses involved in complex mutual interdependencies for aphid transmission such as poleroviruses, umbraviruses and associated satellites, which can be the basis for further investigations of synergistic or antagonistic virus-vector-host relationships.

## Introduction

Agriculture has drastically changed biodiversity at the landscape level by converting natural habitats into intensively managed environments. As a result, an increasing proportion of natural habitats lie adjacent to agricultural lands, creating what is referred to as the agroecological interface, which integrates wild lands as highly fragmented patches within a matrix of crop-, pasture- and grasslands [1]. Such intersections of managed and unmanaged agro(eco)systems can directly influence the emergence of pathogens via pathogen spill-over and spill-back events between the different compartments [1–3]. This is particularly important with regards to viruses that can infect both native wild plants and cultivated crop plants, allowing them to move through the ecological interface [3]. Wild plants can thus play a role as reservoirs of virus infecting cultivated plants. On the other hand, viruses infecting cultivated plants can spread into wild plants and influence their fitness within unmanaged ecosystems in more or less complex or subtle ways [4]. The agroecological interface is therefore an important boundary that can contribute to the structuring of virus communities and thus directly and indirectly influence ecosystem functions. Viruses are ubiquitous biological entities in diverse environments and play a significant role as pathogens in agriculture, where they are estimated to be responsible for half of emerging diseases [5]. However, in some instances and in particular when environmental conditions change, viruses have been suggested to switch from parasitic to mutualistic interactions with their host [6]. Further complicating the picture, in wild plants viruses are often asymptomatic and it has been suggested that one of the reasons for this situation might be viruses long co-evolution with wild hosts and vectors in complex trophic interactions [7].

The family *Apiaceae* comprises about 3,700 species consisting of wild plants and important crops [8]. Carrot (*Daucus carota* L) is the most widely grown crop within the *Apiaceae* family. In total, more than 30 viruses from 12 different families are known to infect carrots [9–11] with some of them causing severe damages to carrot production. Early studies have focused on carrot necrotic dieback virus (CNDBV, genus *Sequivirus*) and the carrot motley dwarf (CMD) complex. CNDBV was originally described as the Anthriscus strain of parsnip yellow fleck virus (PYFV, genus *Sequivirus*) [12] as it naturally infects cow parsley (*Anthriscus sylvestris* L.). In carrots, it causes leaf necrosis, severe stunting and dieback [13]. The CMD complex was first described in Australia by Stubbs [14] and is caused by a mixed infection of a polerovirus, carrot red leaf virus (CtRLV), an umbravirus, carrot mottle virus (CMoV) or carrot mottle mimic virus (CMoMV) and, in some instances, a polerovirus-associated RNA, carrot red leaf virus-associated RNA (CtRLVaRNA) [15]. The umbravirus and the associated RNA lack a capsid protein and their RNAs are transcapsidated by the coinfecting polerovirus for transmission by the aphid *Cavariella aegopodii*. The CMD complex causes leaf reddening and yellowing, stunting and reduces sugar content of taproots [14, 15]. Another economically important virus causing disease in carrot crops is carrot yellow leaf virus (CYLV), a closterovirus, which has been associated with internal root necrosis [16]. Additional novel viruses infecting carrot crops, such as novel members of the *Chordovirus* and *Closterovirus* genera have also been identified [16]. However, less is known about viruses infecting wild carrots which may represent an important reservoir of carrot-infecting viruses.

Recent advances in sequencing technologies such as high-throughput sequencing (HTS) have revolutionized the field of viral ecology over the past decade by moving from a "one host-one pathogen" perspective to analyzing the entire phytovirome at ecosystem level. HTS-based studies have highlighted the importance of wild plants as a source of virus diversity [17–21]. More recent studies have confirmed the importance of unmanaged wild plants surrounding cultivated crop species in the epidemiology, ecology, distribution and emergence of viruses [20, 22–25]. Carrot represents a particularly interesting model plant as the cultivated crop (*Daucus carota* ssp. *sativus*) and its weedy relative, wild carrot (*Daucus carota* ssp. *carota*), belong to the same species and therefore represent a host pair with the lowest expected genetic barrier to virus flow. The aim of the present study was to characterize virus communities in wild and cultivated carrots using a double-stranded RNA (dsRNA)-based metagenomic approach. We have identified a rich and diverse carrot virome in different regions of France and central Spain, providing the basis for further studies on the role of wild plants as virus reservoirs and on the complex interplay and interdependencies of viruses in mixed infections as well as the underlying evolutionary processes in relation to their vectors and hosts.

## Materials and methods

### Sample collection and processing

**Fig 1:**
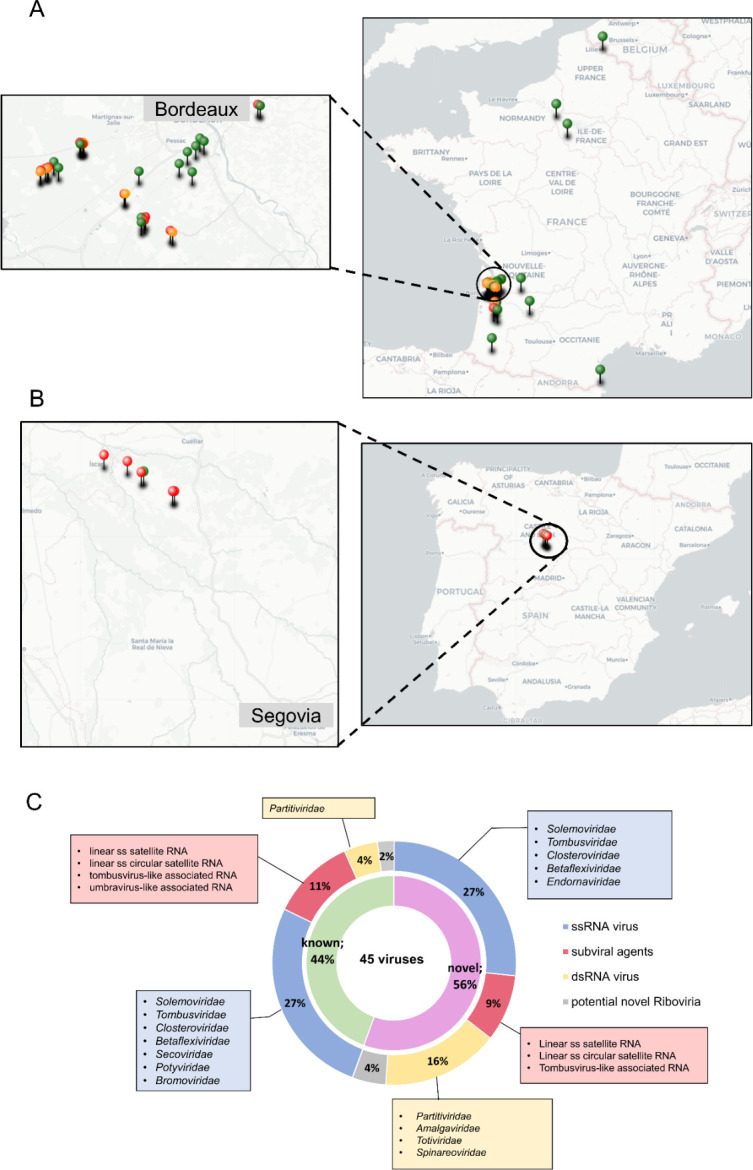
Geographical origin of the sampled carrot populations used in the present study (A-B) and proportion of known and novel viruses identified in their virome, together with the corresponding viral families (C). The maps show sampling locations in France (A) over two years (2019 and 2020) and Spain (2021, B), with green pins corresponding to wild carrot populations and red and orange pins to cultivated and off-type carrot populations respectively (these later two types being hereafter collectively referred to as field carrot populations). Maps were created using framacarte.org.

In July-August 2019, 16 different carrot populations were sampled in south-west France (Gironde and Landes Départements of the Nouvelle Aquitaine region) (S1 Table in S1 File). The sampled carrot populations comprised four cultivated fields, four populations growing within the same cultivated carrot fields and showing some characteristics of wild carrots (early bolting, small and often white tap root) suggesting an origin from seed contaminations of the planted commercial varieties and thereafter referred to as off-type carrots, and eight wild carrot populations growing within a few tens to hundreds square meters. In July-September 2020, carrot populations were sampled across a north south transect of France (S1 Table in S1 File). In total, 29 carrot populations were collected, comprising 11 cultivated carrots populations, three off-type populations and 15 wild populations. A total of 45 carrot populations were therefore sampled in France over the two years (S1 Table in S1 File and Fig 1A). Additional carrot populations were sampled in June 2021 near Segovia (central Spain), including five cultivated and one wild carrot populations (S1 Table in S1 File and Fig 1B).

In each sampled population, leaves from 50 individual plants were collected. Carrot plants were sampled regardless of the presence of viral symptoms, allowing to take into account asymptomatic viral infections, but plants with evident fungal infection or necrosis were excluded. Leaf samples were stored desiccated over anhydrous CaCl_2_ (Sigma-Aldrich, Saint-Quentin-Fallavier, France) until use. For each population, a pool corresponding to the 50 sampled plants was assembled (about 0.1 g leaf material per plant).

### Double-stranded RNAs purification and Illumina sequencing

Double-stranded (ds) RNAs were purified from each pool consisting of 50 carrot plants. Samples were ground to a fine powder using liquid nitrogen and a mortar and pestle and dsRNAs purified by two rounds of CC41 cellulose chromatography as described [26]. A negative extraction control was included by using only buffer. In some cases, dsRNAs were also extracted from individual plants using the same protocol. Purified dsRNAs were converted to cDNA and randomly amplified while simultaneously adding MID tags by RT-PCR [26, 27]. Random amplification PCR products were purified using the MinElute PCR purification kit (Qiagen, Courtaboeuf, France) and their concentration determined spectrophotometrically. Equal amounts of amplification products from each sample/pool were sent for Illumina sequencing in a multiplexed format (2×150 bp) on the NovaSeq 6000 system at the GetPlaGe platform (GenoToul INRAE Toulouse, France).

### Bioinformatic analysis of Illumina sequencing reads: Demultiplexing, quality trimming, *de novo* assembly, contigs annotation and read-mapping

Sequencing reads were demultiplexed and trimmed on quality using CLC Genomic Workbench, version 21.0.3 (CLC-GW, Qiagen) using default settings and a minimum read length of 60 nucleotides (nt). Contigs were assembled *de novo* using a minimal contig length of 250 nt. Viral contigs were annotated by a comparison against the non-redundant sequence (nr) database in GenBank using BlastX [28]. Identified contigs were manually scaffolded, if needed, using the most closely related reference sequence from NCBI GenBank. They were verified and extended by repeated rounds of mapping of residual reads using CLC-GW. When a given virus was identified in multiple samples, the longest contig with the deepest coverage was finally selected to be used as a reference sequence for that virus and was deposited in GenBank. Novel virus species were identified and tentative taxonomic assignations proposed based on the calculation of percent pairwise identities calculated using Mega7 [29] and multiple nucleotide or amino acid alignments with reference sequences selected according to the International Committee on Taxonomy of Viruses (ICTV) species demarcation criteria for each virus genus or family.

In order to determine the geographic distribution of the identified viruses, reads from datasets, normalized to a common depth per sample using the read sampling tool in CLC-GW, were then mapped back to reference contigs (for novel viruses) and reference genomic sequences from GenBank (for known viruses) using CLC-GW and nt identity and length fraction set at a minimum of 90%. The mapped reads were then manually checked to ensure the identity of the detected virus. Two approaches were used to address the well-known HTS inter-sample cross talk issue. The first one considered the viral reads identified for each virus in the blank control for each run. The second one used data from individually analyzed plants for which we also had both virus-specific RT-PCR testing results, so that plants that tested negative in RT-PCR but showing a few reads for a given virus in the corresponding dataset could be used calculate an average number of background reads per million sequencing reads. With this strategy, a separate detection threshold could be calculated for those viruses that were screened by RT-PCR. For viruses not tested by RT-PCR, we used as a threshold the average of the threshold values determined for the RT-PCR tested viruses. Finally, the thresholds obtained for each virus using either the blank controls or the RT-PCR testing were compared and the highest value retained to establish the final HTS positive detection threshold for each virus and clean the reads mapping table for each sample/virus combination. Ultimately, identification of reads for a particular virus above the threshold translates in presence of this virus in the tested carrot population and at the site of that population.

In some cases, genomes were completed by determining 5’ and 3’ genome ends by Rapid Amplification cDNA Ends (RACE) experiments using specific primers (S2 Table in S1 File) and the SMARTer RACE Kit (Takara Bio Europe, Saint-Germain-en-Laye, France), as recommended by the company.

Specific two-step RT-PCR assays [30] were developed for selected novel viruses to identify infected individual plants from positive pools. Primers were designed manually in MEGA 7 using multiple alignments generated from contigs of the corresponding viruses and of related GenBank sequences or of contigs of related viruses, with the aim to design primers that would amplify all isolates of a given virus but be specific enough not to amplify isolates of closely related viruses (S2 Table in S1 File). PCR products were visualized on 1.2% agarose gels and selected amplicons were directly Sanger sequenced (Eurofins Genomics, France) to confirm the results and the specificity of the amplification primers.

### Analysis of viral genomic sequences, phylogenetic and recombination analyses

Open reading frames (ORFs) were predicted using CLC-GW and confirmed using the ExPASy Translate Tool [31]. Multiple nt or aa sequences alignments were performed using ClustalW as implemented in MEGA 7 [32]. Recombination events were analyzed from such multiple alignments using the RDP4 package [33]. Only recombination events detected by at least four out of seven implemented algorithms were considered. Neighbor-joining trees were inferred from whole genomes or conserved gene products multiple alignments and strict nt or aa identities calculated using MEGA 7. The significance of branches was estimated with 1,000 bootstrap replicates.

## Results

During summer 2019 and 2020, a total of 45 different carrot populations were sampled in France, covering a north south country gradient. Additionally, five cultivated and one wild carrot populations were sampled near Segovia (central Spain) in 2021. Details on the sampled carrot populations are shown in S1 Table in S1 File and in Fig 1A and 1B. Illumina sequencing of cDNAs obtained from highly purified dsRNAs prepared from pools of 50 plants for each population and subsequent bioinformatic analyses revealed a rich and diverse virome comprising 45 viruses from 12 different virus families. Paralleling other virome studies, in most cases, long contigs representing near complete genomes with high average coverage could be assembled, demonstrating the feasibility of assembling such long contigs from pools of plants from the same species/location [22, 25]. Twenty of the identified viruses (44%) belong to already known species, while the remaining 25 viruses (56%) represent or possibly represent novel species in nine different viral families, unclassified subviral agents (satellites or associated RNAs) or two putative novel *Riboviria* whose precise higher order taxonomic status remains to be established (Fig 1C). Details of novel and known viruses identified are presented in Tables 1 and 2, respectively. A more detailed description and analysis of the identified viruses is provided in what follows.

**Table 1:** Novel viruses identified in different carrot populations in France and Spain with details on the corresponding sequence library.

Virus Family	Virus genus	virus name	virus acronym	Genome type	Accession number^a^	scaffold length (nt)^b^	mapped reads	average coverage	population type	country
*Solemoviridae*	*Polerovirus*	carrot polerovirus 1	CaPV1	ssRNA+	OP886450*	5671	43652 (0.48%)	873x	wild	France
*Solemoviridae*	*Polerovirus*	carrot polerovirus 2	CaPV2		OP886451	5213	14916 (0.15%)	324x	wild	France
*Solemoviridae*	*Enamovirus*	carrot enamovirus 1	CaEV1		OP886449*	5100	1403 (0.01%)	31x	wild	France
*Tombusviridae*	*Umbravirus*	carrot umbravirus 1	CaUV1		OP886454	3308	88394 (0.63%)	3033	wild	France
*Tombusviridae*	*Umbravirus*	carrot umbravirus 2	CaUV2		OP886452*	4255	22748 (0.24%)	605x	wild	France
*Tombusviridae*	*Umbravirus*	carrot umbravirus 3	CaUV3		OP886453	4059	50896 (0.36%)	1421x	wild	France
*Closteroviridae*	*Closterovirus*	carrot closterovirus 2	CtCV2		OP886455*	16302	3553362 (48.76%)	24536x	cultivated	France
*Closteroviridae*	*Closterovirus*	carrot closterovirus 3	CtCV3		OP886456*	16291	602434 (11.26%)	4173x	cultivated	France
*Betaflexiviridae*	*Chordovirus*	carrot chordovirus 3	CChV3		OP886457	6881	2839 (0.05%)	39x	off-type	France
*Betaflexiviridae*	*Chordovirus*	carrot chordovirus 4	CChV4		OP886458*	7703	1672 (0.02%)	23x	wild	France
*Betaflexiviridae*	*Vitivirus*	carrot vitivirus 1	CaVV1		OP886459*	7546	14828 (0.43%)	221x	wild	France
*Endornaviridae*	*Alphaendornavirus*	carrot alphaendornavirus	CaAEV1		OP886460	14578	80742 (1.87)	608x	wild	France
linear ss satellite RNAs	unclassified	carrot mottle virus satellite RNA 2	CtMoVsatRNA2		OP886481	323	1777 9 (0.01%)	620x	wild	France
tombusvirus-like-associated RNA	unclassified	carrot red leaf virus-associated RNA 2	CtRLVaRNA2		ON603907	[38]				
small circular ss satellite RNAs	unclassified	carrot red leaf virus satellite 1	CtRLV Sat1		OM962993	[9]				
small circular ss satellite RNAs	unclassified	carrot red leaf virus satellite 2	CtRLV Sat2		OM962994	[9]				
*Partitiviridae*	*Deltapartitivirus*	carrot cryptic virus 2	CaCV2	dsRNA	OP886461-62*	3329	932448 (24.19%)	31592x	wild	France
*Partitiviridae*	*Deltapartitivirus*	carot cryptic virus 3	CaCV3		OP886463-64*	3345	408353 (14.99%)	13634x	wild	Spain
*Amalgaviridae*	*Amalgavirus*	carrot amalgavirus 1	CaAV1		OP886466	3223	26411 (0.26%)	929x	wild	France
*Amalgaviridae*	*Amalgavirus*	carrot amalgavirus 2	CaAV2		OP886465	777	130 (0.00%)	19x	wild	Spain
*Reoviridae*	*Reovirus*	carrot reovirus 1	CaRV1		OP886467-76*	26081	2792566 (32.23%)	12089x	wild	France
*Reoviridae*	*Reovirus*	carrot reovirus 2	CaRV2		OP886477-79	3163	4372 (0.10%)	156x	wild	France
*Totiviridae*	*Totivirus*	carrot-associated toti-like virus	CaaTLV		OP886480*	5128	403094 (4.98%)	8876x	wild	France
putative novel Riboviria	putative novel riboviria	carrot flavi-like virus 1	CaFLV1	ssRNA?	OM681407	[47]				
putative novel Riboviria	putative novel riboviria	carrot Ker-like virus	CaKLV	ssRNA?		to be published elsewhere (TC, AM, DS et al., manuscript in preparation)				

a: viral sequences deposited in GenBank and corresponding to a complete genome or, at least, to a complete coding potential are indicated by an asterisk

b: for viruses with divided genomes, the cumulated genome length is indicated

**Table 2:** Known viruses identified in different carrot populations in France and Spain.

Virus Family	Virus genus	Virus name	Acronym	Genome nucleic acid	Accession number
*Solemoviridae*	*Polerovirus*	carrot red leaf virus	CtRLV	+ ssRNA	NC_006265
*Solemoviridae*	*Polerovirus*	wild carrot red leaf virus	WCtRLV		LT615231
*Tombusviridae*	*Umbravirus*	carrot mottle virus	CMoV		KF533714
*Tombusviridae*	*Umbravirus*	wild carrot mottle virus	WCMoV		LT615232
*Tombusviridae*	*Umbravirus*	carrot mottle mimic virus	CMoMV		NC_001726
*Closteroviridae*	*Closterovirus*	carrot closterovirus 1	CtCV1		KF533697
*Betaflexiviridae*	*Closterovirus*	carrot yellow leaf virus	CYLV		KF533699
*Betaflexiviridae*	*Chordovirus*	carrot Chordovirus 1	CChoV1		NC_025469
*Betaflexiviridae*	*Chordovirus*	carrot Chordovirus 2	CChoV2		NC_025468
*Secoviridae*	*Torradovirus*	carrot Torradovirus 1	CaTV1		NC_025479 and NC_025480
*Potyviridae*	*Potyvirus*	apium virus Y	AVY		NC_014905
*Bromoviridae*	*Ilarvirus*	solanum nigrum ilarvirus 1	SnIV1		MN216370
linear ss satellite RNAs	*unclassified*	carrot mottle virus satellite RNA	CMoVsatRNA		NC_030649
Tombusvirus like associated RNA	*unclassified*	carrot red leaf virus associated RNA	CtRLVaRNA		NC_003871
Tombusvirus like associated RNA	*unclassified*	arracacha latent virus E associated RNA	ALVEaRNA		MF136436
Tombusvirus like associated RNA	*unclassified*	beet western yellows virus associated RNA	BWYVaRNA		KF533709
Umbra-like associated RNA	*unclassified*	parsley umbravirus 1	ParUV1		OM419177.1
*Partitiviridae*	*Alphapartitivirus*	carrot cryptic virus	CaCV1	dsRNA	NC_038824 and NC_038823
*Partitiviridae*	*Betapartitivirus*	dill cryptic virus 2	DiCV2		NC_021147 and NC_021148
*putative novel Riboviria*	*putative novel riboviria*	carrot-associated RNA virus 1	CaRNAV1	ssRNA?	OM419188

### Discovery of different known and novel umbraviruses and of a potential novel class of umbravirus-like virus

**Fig 2:**
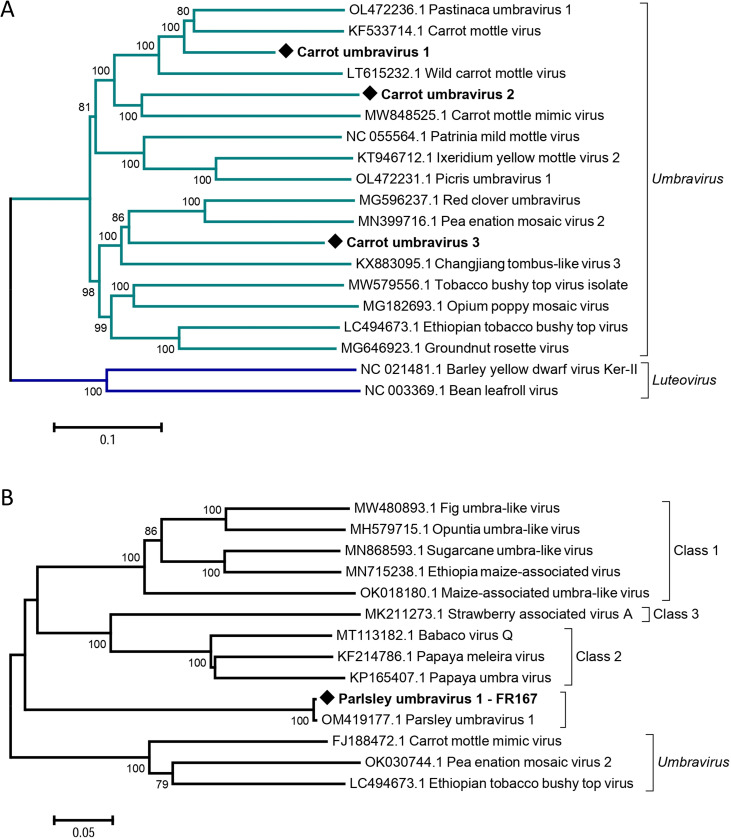
Neighbour-joining trees reconstructed from full-length nucleotide alignments of the RdRp gene from identified umbraviruses and related reference sequences from GenBank (A) and from partial RdRp gene sequences from umbravirus-like-associated RNAs (B). Bootstrap values above 70% are shown (1,000 replicates). The scale bars represent 10% (A) and 5% (B) nucleotide divergence between isolates. Accession numbers are indicated for each reference sequence and novel or tentatively novel viruses are indicated by black diamonds. The corresponding genus or group is indicated to the right of each bracket.

The French and Spanish carrot viromes were found to be rich in viruses of the genus *Umbravirus*. All three umbraviruses known to infect carrots, including carrot mottle virus (CMoV), carrot mottle mimic virus (CMoMV) and the more recently reported wild carrot mottle virus (WCMoV) have been identified in the three different carrot population types (cultivated, off-type and wild). They were detected in more than 90% of all sampled populations in France and Spain. In addition, we identified in different French cultivated and wild populations a virus of uncertain taxonomic status related to CMoV and referred to here as carrot umbravirus 1 (CaUV1), and two novel umbraviruses, carrot umbravirus 2 (CaUV2) and carrot umbravirus 3 (CaUV3). In all cases, the genome organization was typical for the genus with four ORFs coding respectively from 5’ to 3’ for a replicase-associated protein, a RNA-dependent RNA polymerase (RdRp, expressed as a fusion as a consequence of a -1 ribosomal frameshift) and long distance movement and cell to cell movement proteins encoded by overlapping 3’ ORFs. A phylogenetic tree based on a multiple nt alignment of the RdRp genes for known umbraviruses and for those novel ones reported here is shown in Fig 2A.

A 3.3kb scaffold with >3,000-fold coverage could be obtained for CaUV1, covering an estimated 79% of the full genome. This sequence shows a pairwise nt identity of 78% and 76% to pastinaca umbravirus 1 (PasUV1, GenBank OL472236) and CMoV (GenBank KF533714), respectively, in its partial RdRp gene (S3 Table in S1 File), which is above the ICTV species demarcation threshold of 70% nt identity between virus species for the *Umbravirus* genus. However, the available CaUV1 sequence covers only 67% of the full RdRp gene and it is therefore not possible to estimate the identity percentage that would be reached if full genomes were compared and, therefore, to decide whether CaUV1 should be regarded as a variant of PasUV1 or CMoV or as belonging to a new umbravirus species. A recombination analysis performed using the multiple nt sequence alignment used for phylogenetic analysis suggested the existence of a recombination event linking PasUV1, CMoV and CaUV1. Details of this recombination event, detected by all algorithms and with a combined Bonferoni corrected probability of 1.7xE^-35^ are shown in S1 Fig. This event involves the 3’ half of the movement protein (MP) gene and the 3’ non-coding region (3’NCR), with up to 98% nt identity in this ca. 1 kb region between CMoV (KF533714) and CaUV1. It is however not possible to decide whether CaUV1 or CMoV should be considered as the parent or the recombinant, since it is not possible to orient this specific recombination event. CaUV1 was detected in all three carrot population types, eventhough the proportion of populations with CaUV1 infection was slightly higher for cultivated (8/15, 53.3%) and off-type carrots (4/7, 57.1%) than for wild ones (7/23, 30.1%).

Nearly complete genomes of the other two novel umbraviruses, CaUV2 and CaUV3 could be assembled of respectively 4.25 and 4.06 kb and 605x and 1421x average coverage. These assembled genomes show highest nt pairwise identities of respectively 64% and 61% with carrot mottle mimic virus (CMoMV, GenBank MW848525) and red clover umbravirus (RCUV, GenBank MG596237), based on full genome alignments (S3 Table in S1 File) and only 47% nt identity with each other. These identity values are clearly below the 70% nt identity cut-off threshold in the *Umbravirus* genus, supporting the notion that the sequenced isolates belong to distinct novel species. Both viruses were detected in more than half of the wild populations over the two sampling years in France (65.21%, 15/23) and in a smaller proportion of field carrot populations. CaUV2 was detected in 33.3% (5/15) of cultivated and 42.8% (3/7) of off-type ones, while the corresponding values for CaUV3 were respectively 13.3% (2/15) and 57.1% (4/7). These two novel umbraviruses were not detected in any of the six Spanish carrot populations.

A fourth partial umbra-like sequence was identified in a total of seven French carrot populations: one wild and one off-type 2019 populations (FR19-2 and FR19-3, S1 Table in S1 File) and two cultivated, one off-type and two wild 2020 populations (FR20-2, FR20-4, FR20-6, FR20-15 and FR20-25). The agent was also detected in a cultivated Spanish population and in the wild population sampled on the same site (ES21-4 and ES21-5, S1 Table in S1 File). The assembled partial genomic scaffold of 2.3kb with a internal gap estimated at ca. 370nt showed 97% nt identity in a BLASTN search with the recently reported parsley umbravirus 1 (PaUV1, OM419177; 3,087nt). However, despite the PaUV1 name, this virus does not show phylogenetic affinities or a genomic organization typical of umbraviruses. In particular, only three ORFs have been predicted for PaUV1, similar to class 2 umbravirus-like-associated RNAs [34] and its phylogenetic clustering, based on a the partial RdRP gene (OM419177, nt 1140–2157) shows that it clusters away from umbraviruses and forms a distinct cluster within the umbra-like-associated RNAs (Fig 2B) suggesting that this PaUV1 may represent a distinct umbravirus-like-associated RNAs.

### Identification of known and novel viruses of the family *Solemoviridae*

Carrot red leaf virus (CtRLV) is a well-known carrot-infecting polerovirus that is part of the complex responsible for the CMD disease affecting carrot crops. CtRLV was detected in most of the sampled French populations (41/45, 91%) and in all Spanish cultivated populations (5/5, 100%), but not the wild one. The sequence of a distinct polerovirus, tentatively named wild carrot red leaf virus was deposited in 2020 in GenBank (LT615231). This virus was found exclusively in wild carrot populations, infecting 17/23 of wild populations sampled over two years in France. It was not detected in any of the sampled Spanish populations. A novel enamovirus, tentatively named carrot enamovirus 1 (CaEV1) and two novel poleroviruses tentatively named carrot polerovirus 1 (CaPV1) and carrot polerovirus 2 (CaPV2) were additionally discovered in various French carrot populations (Table 1).

**Fig 3:**
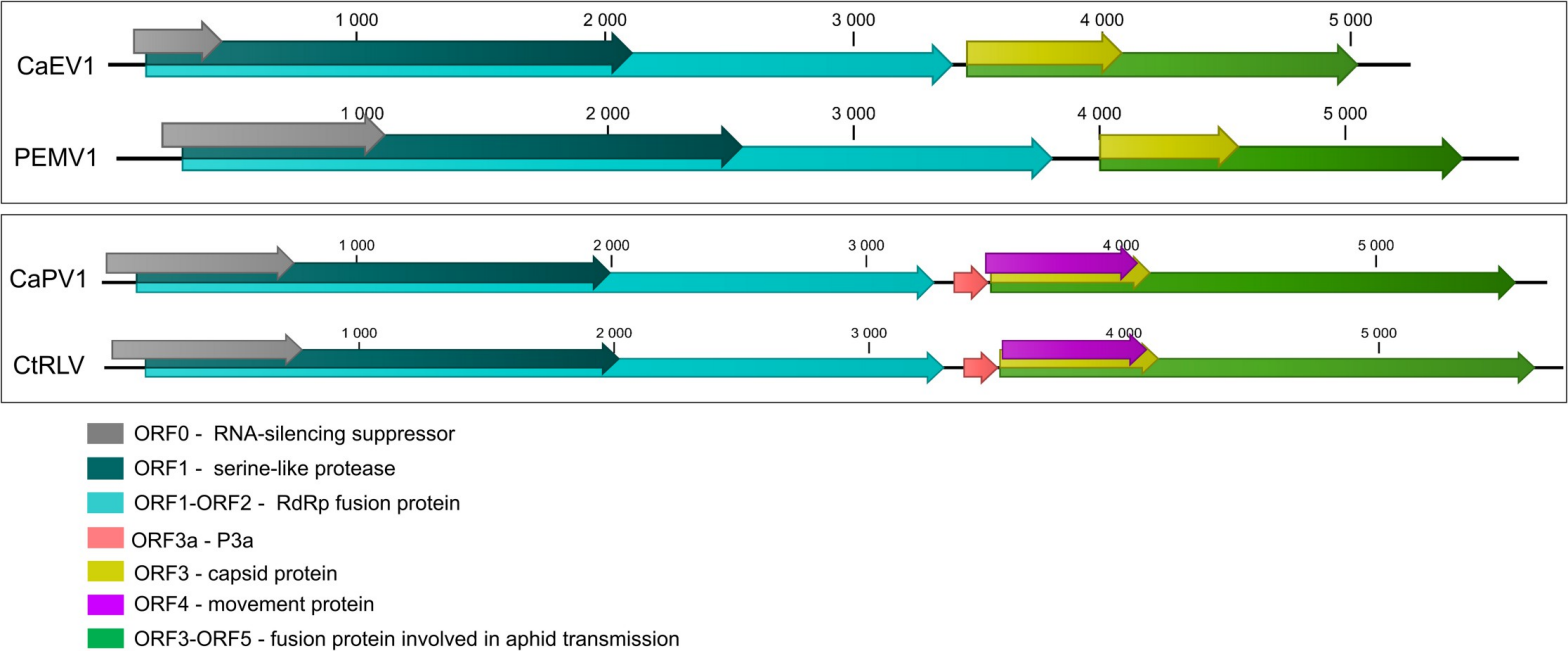
Schematic representation of the genome organization of the novel viruses carrot enamovirus 1 (CaEV1) and carrot polerovirus 1 (CaPV1). The genomes of pea enation mosaic virus 1 (PEMV1, *Enamovirus*, GenBank MN497824) and of carrot red leaf virus (CtRLV, *Polerovirus*, GenBank LC434063) are presented for comparison. Open reading frames are indicated by differently coloured arrows and their protein function described in the colour code.

**Fig 4:**
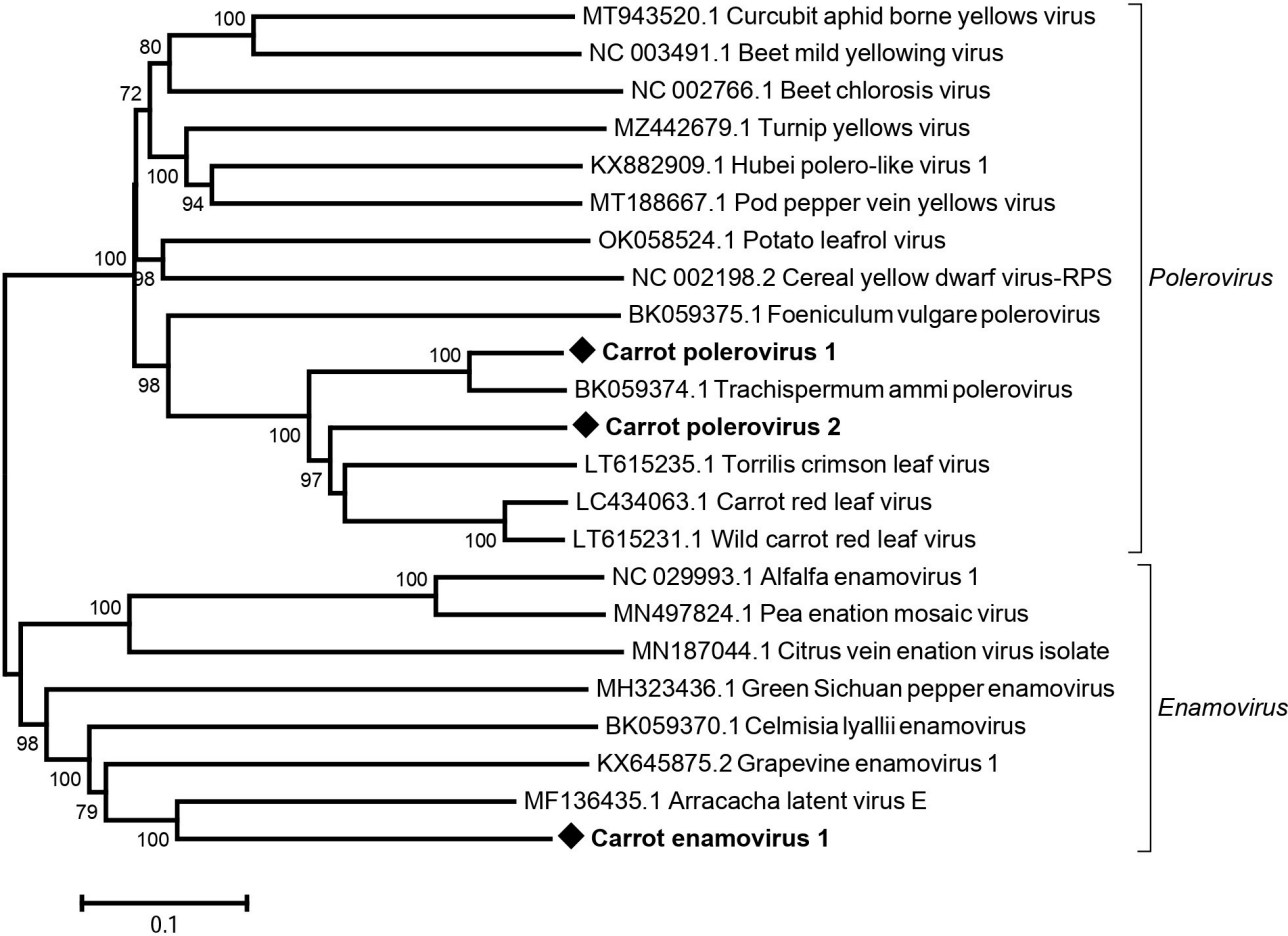
Neighbour-joining tree reconstructed from amino acid alignment of the full length RNA-dependent RNA polymerase from known and novel members of the *Polerovirus* and *Enamovirus* genera identified and of related reference sequences from GenBank. Bootstrap values above 70% are shown (1,000 replicates). The scale bar represents 10% amino acid divergence between sequences. Accession numbers are indicated for each reference sequence and novel viruses are indicated by black diamonds. The corresponding genus is indicated to the right of each bracket.

Following the initial identification of CaPV1 using the sequencing reads from a wild population sampled in France in 2019 (FR19-15, S1 Table in S1 File), plants from that population were individually tested by specific RT-PCR. Three out of the tested 50 plants were positive for CaPV1 and dsRNAs were purified from one of them and sequenced by Illumina. A complete genome could be assembled from this single-plant dataset and the 5’ and 3’ terminal regions were determined using specific primers in RACE experiments (S2 Table in S1 File). The full genome consists of 5,671 nt (average coverage 873x) and shows a genome organization typical for the genus *Polerovirus* with seven predicted ORFs encoding proteins P0 to P5 as well as a P3a (Fig 3). In Blast searches, CaPV1 is most closely related to Trachyspermum ammi polerovirus with 89% aa identity in the RdRp and 47–69% to other poleroviruses (Fig 4 and S4 Table in S1 File). However, identity levels with other poleroviruses, including Trachyspermum ammi polerovirus are below 70% for the CP, P4 and P5 proteins. CaPV1 was detected in a high proportion (19/23, 82.6%) of the French wild carrot populations over the two sampling years, irrespective of their geographical origin since it was detected in populations sampled either in the north or south of France (S5 Table in S1 File). On the other hand, it was not detected in the field French populations or in the Spanish carrot populations.

A very small contig of ca. 340 nt originally identified from a wild carrot population (FR19-15, S1 Table in S1 File) suggested the existence of an additional, distinct polerovirus. In order to obtain a complete genomic sequence, the same strategy as for CaPV1 was followed: plants of the identified population were individually tested using a specific RT-PCR-assay (S2 Table in S1 File) and the single positive plant used to extract dsRNAs that were then analyzed by HTS. The sequencing reads thus obtained allowed to assemble a near complete genome of 5.2 kb (324x average coverage) but no specific efforts were made to determine the precise missing 5’ and 3’ genome ends. CaPV2 is most closely related to Torrilis crimson leaf virus (GenBank LT615235) and CtRLV (GenBank LC434063) (Fig 4) sharing with them respectively 72% and 71% aa identity in the RdRp (S4 Table in S1 File), values that are well under the 10% aa divergence species cut-off. CaPV2 was identified in one cultivated, one off-type population and in 12 wild French populations but was not identified in Spain. Similar to CaPV1, CaPV2 showed a wide geographical distribution in France, being detected in all five regions from where carrot populations were sampled (S5 Table in S1 File).

Recombination events have been identified between members of the *Polerovirus* genus based on full length genome alignments. Among the viruses identified in the carrot virome, the clearest recombination event, which was identified by all algorithms of the RDP4 program, concerns wild carrot red leaf virus. WCtRLV has been identified as recombinant (combined corrected probability 1.2xE^-113^) with CtRLV as major parent and CaPV1 as minor parent, the recombined region concerning part of the CP readthrough (CP-RT) domain (S1 Fig). Another potential recombination event was tentatively identified by six out of seven algorithms for Trachyspermum ammi polerovirus 1, resulting from a recombination with CaPV1 as major parent and Torrilis crimson leaf virus as minor parent. This tentative recombination event was identified in the CP gene, but with a much lower combined corrected probability of 6.9E^-9^. A summary of both recombination events is given in S1 Fig.

A nearly complete genome of a novel enamovirus, CaEV1, of 5.1 kb (average coverage 31x, Table 1) was obtained from a wild carrot population sampled in France in 2019 (FR19_16, S1 Table in S1 File). The sequence is most closely related to a partial sequence of Arracacha latent virus E (GenBank MF136435, Fig 4) with respectively 57% aa identity and 56% nt identity in the full RdRp and the nearly full-length genome (S4 Table in S1 File). The genome organization of CaEV-1 is typical for the genus, with five predicted ORFs (Fig 3). The first ORF, ORF0, encodes the P0 protein, which has been shown to act as an RNA silencing suppressor [35]. The second ORF, ORF1, harbors a conserved serine-like protease motif (Peptidase S39, Pfam PF02122). The third ORF, ORF2, is translated by a -1 ribosomal frameshift of ORF1, resulting in an ORF1-ORF2 fusion protein, P1-P2, which encodes the viral RdRp. The fourth and fifth ORFs, ORF3 and ORF4, respectively encode the CP (with a conserved luteovirus CP motif, pfam PF00894) and the CP-RT expressed by readthrough of the ORF3 stop codon, contains a pfam PF01690 conserved motif and is thought to be involved in aphid transmission [36]. CaEV1 lacks a movement protein, which is typical for members of the *Enamovirus* genus. It was found more prevalent in wild carrot populations, being detected in 11/23 wild populations but in only 2/15 cultivated populations and one off-type population from France. It was not detected in Spanish carrot populations. In France, it was geographically limited to southwest of France including Nouvelle Aquitaine and one population sampled in the region Occitanie (S5 Table in S1 File).

Several polerovirus-associated RNAs have been identified in the French and Spanish carrots virome including carrot red leaf virus-associated RNA (CtRLVaRNA), beet western yellows virus-associated RNA (BWYVaRNA), and two other such molecules detected for the first time in carrots, arracacha latent virus E-associated RNA (ALVEaRNA) and a new RNA, carrot red leaf virus-associated RNA 2 (CtRLVaRNA-2). These self-replicating subviral agents depend on helper viruses of the family *Solemoviridae* for aphid transmission and have recently been classified as tombus-like associated RNAs (tlaRNAs) due to their phylogenetic affinities with members of the *Tombusviridae* family [37]. A detailed description of their diversity, distribution and preferential association with either wild or cultivated carrots within this metagenomic study has recently been published [38]. Moreover, two novel hammerhead ribozyme containing single-stranded circular satellite RNAs, carrot red leaf virus satellite 1 (CtRLV Sat1) and carrot red leaf virus satellite 2 (CtRLV Sat2) have also been identified in Spanish and French carrot populations and reported in detail recently [39].

Two linear single-stranded satellite RNAs of respectively 745 and 748 nt and named carrot mottle virus satellite RNA (CMoVsatRNA) and carrot mottle mimic virus satellite RNA (CMoMVsatRNA) are known [40]. These two satellites are closely related, since they show 94% nt sequence identity with each other. Given the diversity of these two satellites and their very close relationship, it was not possible to easily distinguish them and, for the sake of simplicity, all related contigs identified in the present study were annotated as representing CMoVsatRNA. It was identified from most of the French populations (41/44, 93%) and all Spanish populations. In addition, smaller contigs of ~320 nt (average coverage of the retained reference contig 620x) were identified from various French populations, showing a more distant relationship to CMoVsatRNA, with only 84–85% nt identity in BLASTN searches against CMoVsatRNA and CMoMVsatRNA. These contigs are therefore tentatively considered as representing a distinct satellite RNA, for which the name carrot mottle virus satellite RNA 2 (CMoVsatRNA2) is proposed. This additional satellite was preferentially identified in French wild carrot populations (91%, 21/23) as compared to cultivated (27%, 4/15) or off-type (43%, 3/7) ones. It was identified in three regions of France (S5 Table in S1 File) but not in Spain.

### Known and novel *Betaflexiviridae* members in the carrot virome

Two chordoviruses, carrot chordovirus 1 (CChV1) and carrot chordovirus 2 (CChV2) have recently been described from carrot samples in the UK [16]. Both viruses were identified at low frequency in the French carrots virome (CChV1 in one cultivated, one off-type and one wild population; CChV2 in two cultivated and two wild populations).

**Fig 5:**
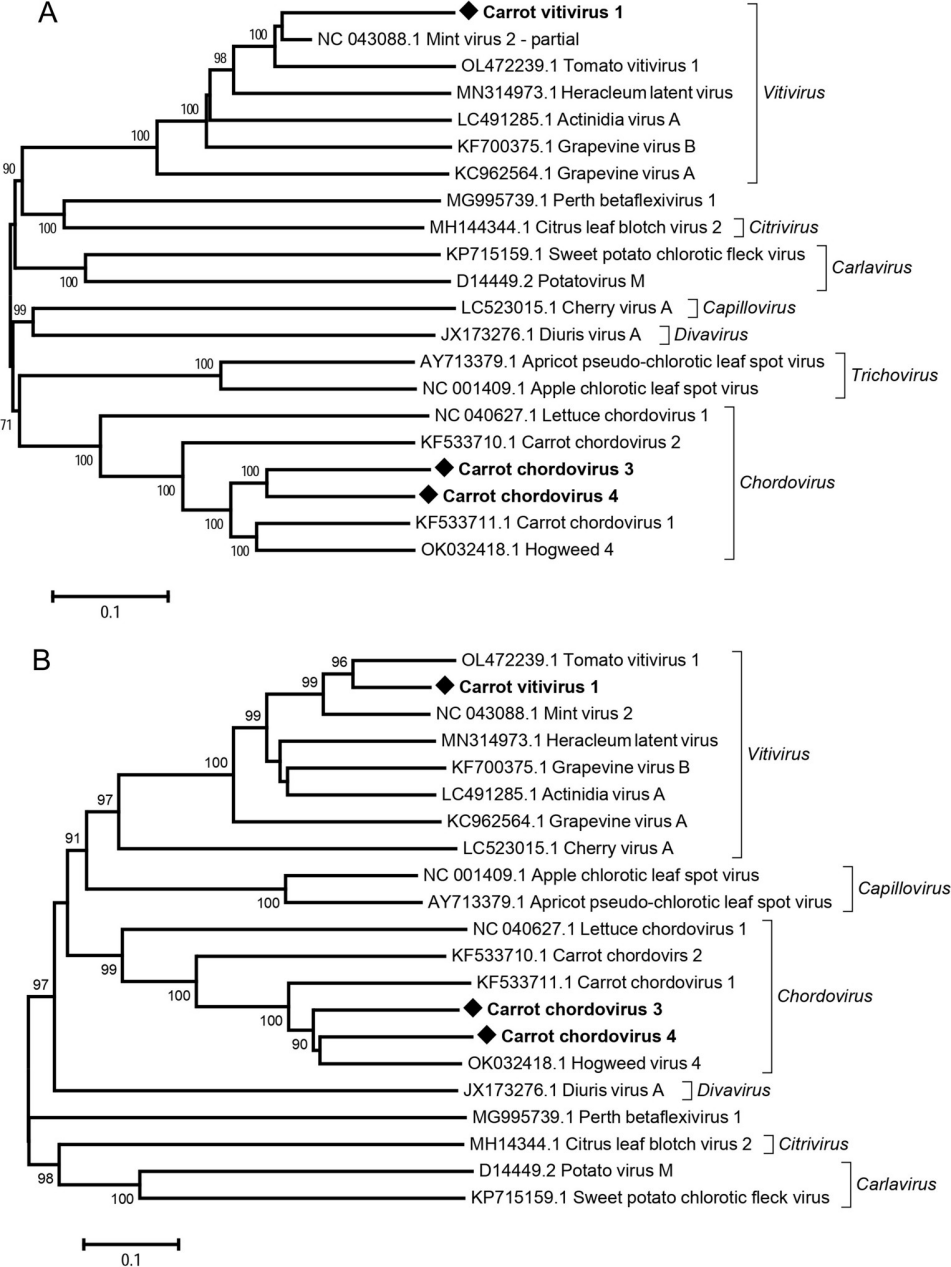
Neighbour-joining trees reconstructed from amino acid alignments of the RNA-dependent RNA polymerase (A) and the capsid protein of novel viruses in the family *Betaflexiviridae* identified in the French carrot virome, the most related reference sequences in GenBank and representative members of each genus. Bootstrap values above 70% (1000x replicates) are shown. The scale bars represent 10% amino acid divergence between sequences. Novel viruses are indicated by black diamonds and accession numbers are given for each reference sequence. Genera are indicated in italics at the right of the trees.

**Fig 6:**
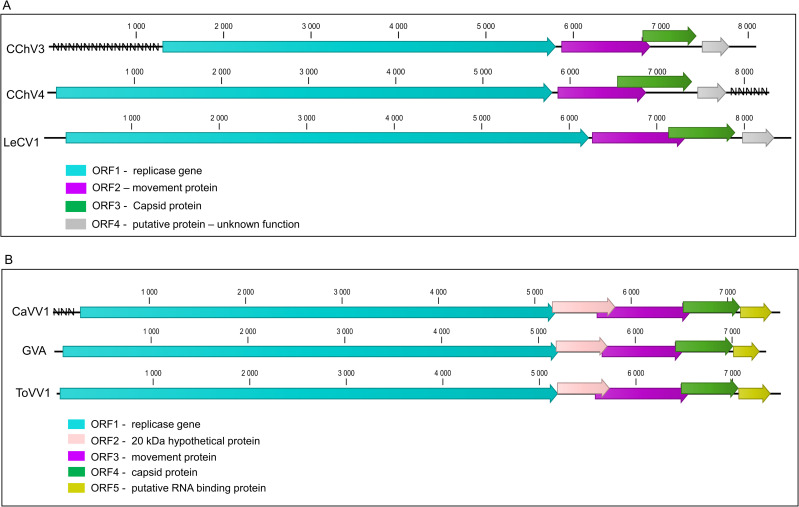
Schematic representation of the genome organization of selected chordoviruses (A) and selected vitiviruses (B). Viruses included in (A) are the novel carrot chordoviruses 3 and 4 (CChoV 3 and 4) and lettuce chordovirus 1 (LeCV1, GenBank NC_040627). Viruses included in (B) are the novel carrot vitivirus 1 (CaVV1), grapevine virus A (GVA, GenBank KC962564) and tomato vitivirus 1 (ToVV1, GenBank OL472239). Missing sequences in the novel genomes reported here are indicated by Ns at the corresponding genome location. Predicted open reading frames are indicated by arrows of different colours and the function of the encoded protein given in the colour code.

In addition, two novel chordoviruses, for which the names carrot chordovirus 3 (CChV3) and carrot chordovirus 4 (CChV4) are proposed, have been identified in one off-type population (FR20-8, S1 Table in S1 File) and one wild population (FR20-16), respectively. A 6,860 nt scaffold (39x average coverage) was obtained for CChV3 covering ~84% of the full genome. In GenBank BlastN searches, this contig shows highest homology to hogweed virus 4 (GenBank OK032418). Pairwise comparisons of CChV3 with the closest related sequences from GenBank show highest pairwise aa identities of 67% and 69% to hogweed virus 4 in the RdRp and CP gene, respectively (S6 Table in S1 File and Fig 5A and 5B), which are well below the 80% aa identity species cut-off thresholds for these proteins in the *Betaflexiviridae*. CChV4, identified as a 7.7 kb nearly complete genome with 23x average coverage (Table 1), also shows highest aa pairwise identities to hogweed virus 4, with respectively 71% and 69% in the RdRp and CP. Similar to lettuce chordovirus 1, the two novel chordoviruses show four predicted ORFs, encoding from 5’ to 3’ a large, ca. 210 kDa replicase (REP), a 30K-family movement protein (Pfam PF01107), a capsid protein (Pfam PF05892) and a fourth protein (12.3 kDa) of unknown function (Fig 6A). CChV3 and CChV4 share with each other respectively 73% and 69% aa identy in the RdRp and CP and cluster together in the corresponding reconstructed phylogenetic trees (Fig 5A and 5B and S6 Table in S1 File).

A tentative novel vitivirus, carrot vitivirus 1 (CaVV1), was identified in a single population of wild carrots sampled in 2020 in Gironde, France (FR20-20, S1 Table in S1 File). This is, to our knowledge the first report of a member of the genus *Vitivirus* infecting carrots. A near complete CaVV1 genome scaffold of 7.5 kb with an average coverage of 221x was reconstructed (Table 1). It shares the highest identity with a partial sequence of mint virus 2 (MV-2, GenBank NC_043088) with 83% aa identity (72% nt identity) in the RdRp (S6 Table in S1 File). While this value is above the species demarcation criterion, the partial MV-2 RdRp sequence comprises only ~30% of the REP, so that it is not possible to estimate overall REP relatedness, but it is estimated to be lower than the computed 83%, since the MV-2 sequence covers the most conserved part of the REP protein. The pairwise aa distance in the CP between CaVV1 and MV-2 is 78%, below the species cut-off threshold. The next closest virus in the pairwise comparisons is tomato vitivirus 1 (GenBank OL472239) with respectively 70% and 81% aa identity in the RdRp and CP (S6 Table in S1 File). CaVV1 shows a typical vitivirus genome organization including five ORFs encoding from 5’ to 3’ a replicase, a 20K-protein of unknown function typical of genus members, a movement protein, a capsid protein and a nucleotide binding protein (Fig 6B). Interestingly, CaVV1 ORF2 does not have a canonical AUG codon but could be expressed from an alternative CUG start codon.

### Known and novel closteroviruses in the carrot virome

**Fig 7:**
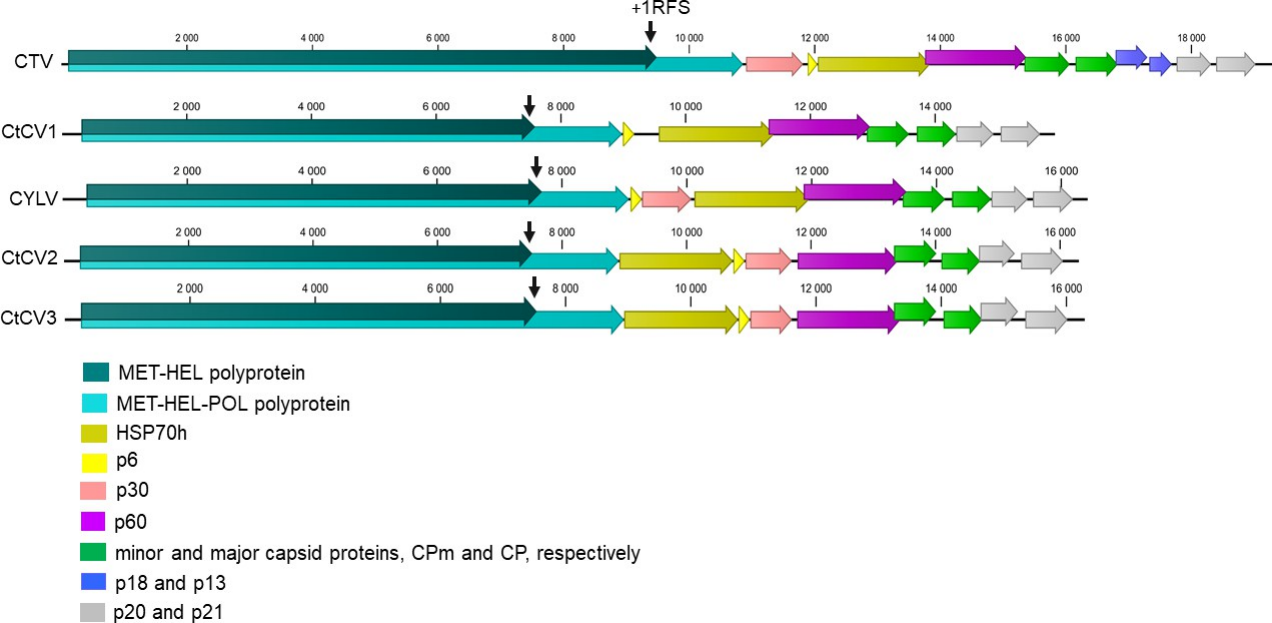
Schematic representation of the genomic organization of known and novel *Closterovirus* genus members. Viruses included are citrus tristeza virus (CTV), carrot closterovirus 1 (CtCV1), carrot yellow leaf virus (CYLV), and the novel carrot closteroviruses 2 and 3 (CtCV2 and CtCV3). ORFs are indicated as arrows in different colours and their function described in the colour code. Vertical arrows indicate putative +1 ribosomal frameshifts (+1RFS).

Carrot yellow leaf virus (CYLV), a known member of the *Closterovirus* genus, was identified in one cultivated population sampled in 2019 in Gironde (FR19-10, S1 Table in S1 File) and in one wild population in the north of France (FR20-22). Carrot closterovirus 1 (CtCV1), previously described in the UK [16] was also identified in the same wild carrot population from the north of France. Two additional novel viruses of the *Closterovirus* genus were identified from a few French or Spanish carrot populations. A large contig covering a nearly complete genome (16.3 kb, average coverage 24,536x, Table 1) was obtained from a French cultivated population (FR20-9, S1 Table in S1 File) and corresponds to a novel closterovirus for which the name carrot closterovirus 2 (CtCV2) is proposed. A second large contig of 14.6 kb was initially assembled from a different cultivated population (FR20-11) sampled in 2020 in France. The contig was extended by repeated mapping of residual reads to obtain a near complete genome scaffold of 16.3 kb (4,173x average coverage, Table 1) for a second novel closterovirus, for which the name carrot closterovirus 3 (CtCV3) is proposed. CtCV3 was identified in two French populations, one cultivated from Nouvelle Aquitaine and one wild from Ile-de-France (FR20-11 and FR20-16, S1 Table in S1 File), and in four cultivated Spanish populations (ES21-1B, ES21-2, ES21-3 and ES21-5, S1 Table in S1 File). These two novel closteroviruses show a genomic organization with 10 predicted ORFs typical for the genus. The 5’ ORFs 1a and 1b encode replication associated proteins: P1a contains RNA methyltransferase (MET) and RNA helicase (HEL) domains, while P1b, expressed by a +1 ribosomal frameshift of ORF1a encodes RdRp. The 3’ -proximal ORFs are expressed from monocistronic subgenomic (sg) RNAs with five downstream genes that are highly conserved among *Closteroviridae* members [41] including a ~6 kDa hydrophobic protein (p6), a HSP70 homolog (HSP70h), a ~60 kDa protein (p60); the minor, duplicate capsid protein (CPd), and the major capsid protein (CP). The two novel closteroviruses, CtCV2 and CtCV3, encode an additional ~30 kDa protein of unknown function which is also present in the CYLV genome and in a few other closteroviruses, such as citrus tristeza virus (CTV, GenBank U16304) or beet yellow stunt virus (BYSV, GenBank U51931) (Fig 7). This gene is however apparently absent from the genome of CtCV1, which presents a more typical genomic organization. A unique feature of CYLV, CtCV2 and CtCV3 is the location of the p30 ORF, which is found upstream the p6 ORF, whereas in CTV and BYSV, it is found directly downstream of ORF1b (Fig 7). In addition, a unique genomic feature of the two novel closteroviruses is the position of the HSP70h ORF, which is located directly downstream the ORF1b, when in all other closteroviruses, it is located downstream the p6 ORF (Fig 7). Lastly, the genomes of the two novel closteroviruses contain two 3’ terminal ORFs encoding respectively proteins of 21 and 20 kDa whose homologs in BYSV have been shown to be involved in systemic movement and suppression of RNA silencing, respectively [41]

The two novel closteroviruses share respectively 83% and 61% aa identity in their RdRp and in the full ORF1a-ORF1b fusion protein, respectively. Pairwise comparisons in the HSP70h and the CP show respectively 80% and 70% aa identity between CtCV2 and CtCV3 (S7 Table in S1 File). Both viruses share 74–78% ORF1a-ORF1b fusion protein aa identity, 65–68% HSP70h aa identity and 44–47% CP aa identity CtCV1 and CYLV. Even though the pairwise distances in the RdRp and HSP70h gene show that the two novel closteroviruses are closely related, their distance is above the ICTV species demarcation threshold of 25% aa divergence in the CP. Altogether, these comparisons suggest that the assembled genomes correspond to viruses that belong to two distinct and novel Closterovirus species.

### A new alphaendornavirus in a wild carrot population

**Fig 8:**
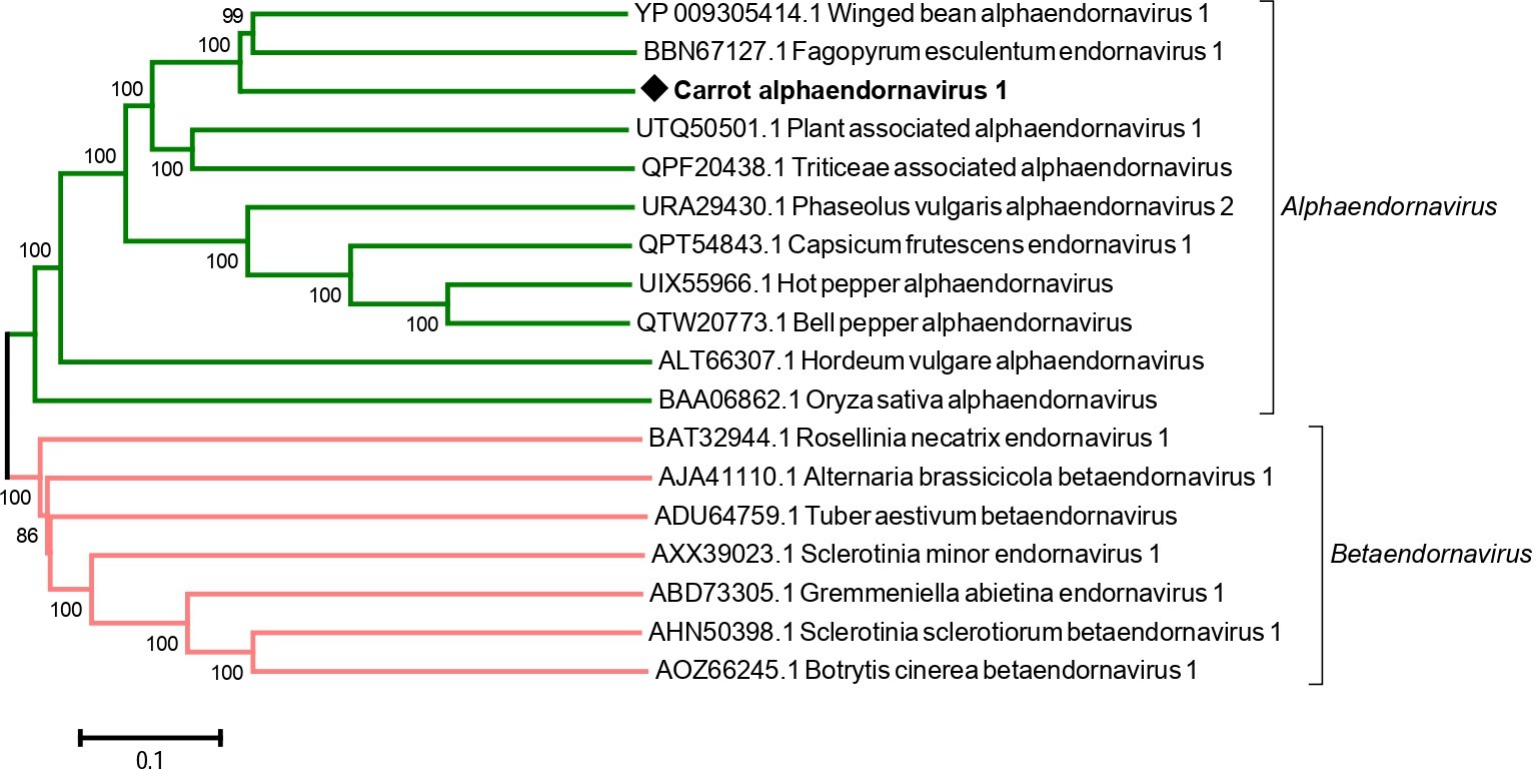
Neighbour-joining tree reconstructed from the amino acid alignment of the RNA-dependent RNA polymerase of the novel carrot alphaendornavirus 1 and of reference sequences of members in the *Alphaendornavirus* and *Betaendornavirus* genera retrieved from GenBank. Carrot alphaendornavirus 1 is indicated by a black diamond. Bootstrap values above 70% (1,000x replicates) are shown. The scale bar represents 10% amino acid divergence between sequences. Accession numbers are given for each reference sequence. Genera are indicated in italics on the right of lateral brackets.

A novel alphaendornavirus was identified from a wild carrot population sampled in the north of France in 2020 (FR20-18, S1 Table in S1 File). According to our knowledge, this is the first record of a virus of the *Endornaviridae* family from carrots. The assembled genomic scaffold of 14.6 kb (average coverage 608x, Table 1) represents a near complete genome and shows highest phylogenetic affinities to winged bean alphaendornavirus 1 (WBEV1, GenBank NC_031336) and fagopyrum esculentum endornavirus 1 (FeEV1, GenBank LC500285) with 54–55% pairwise nt identity and 44–45% pairwise aa identity over the genome polyprotein (S8 Table in S1 File). CaAEV1 clusters together with these two viruses and with related members of the genus *Alphaendornavirus*, separately from members of the *Betaendornavirus* genus but its divergence level with WBEV1 and FeEV1 unmistakeably makes it a novel species (Fig 8).

### Novel *Spinareoviridae* identified in wild carrot populations in France

**Fig 9:**
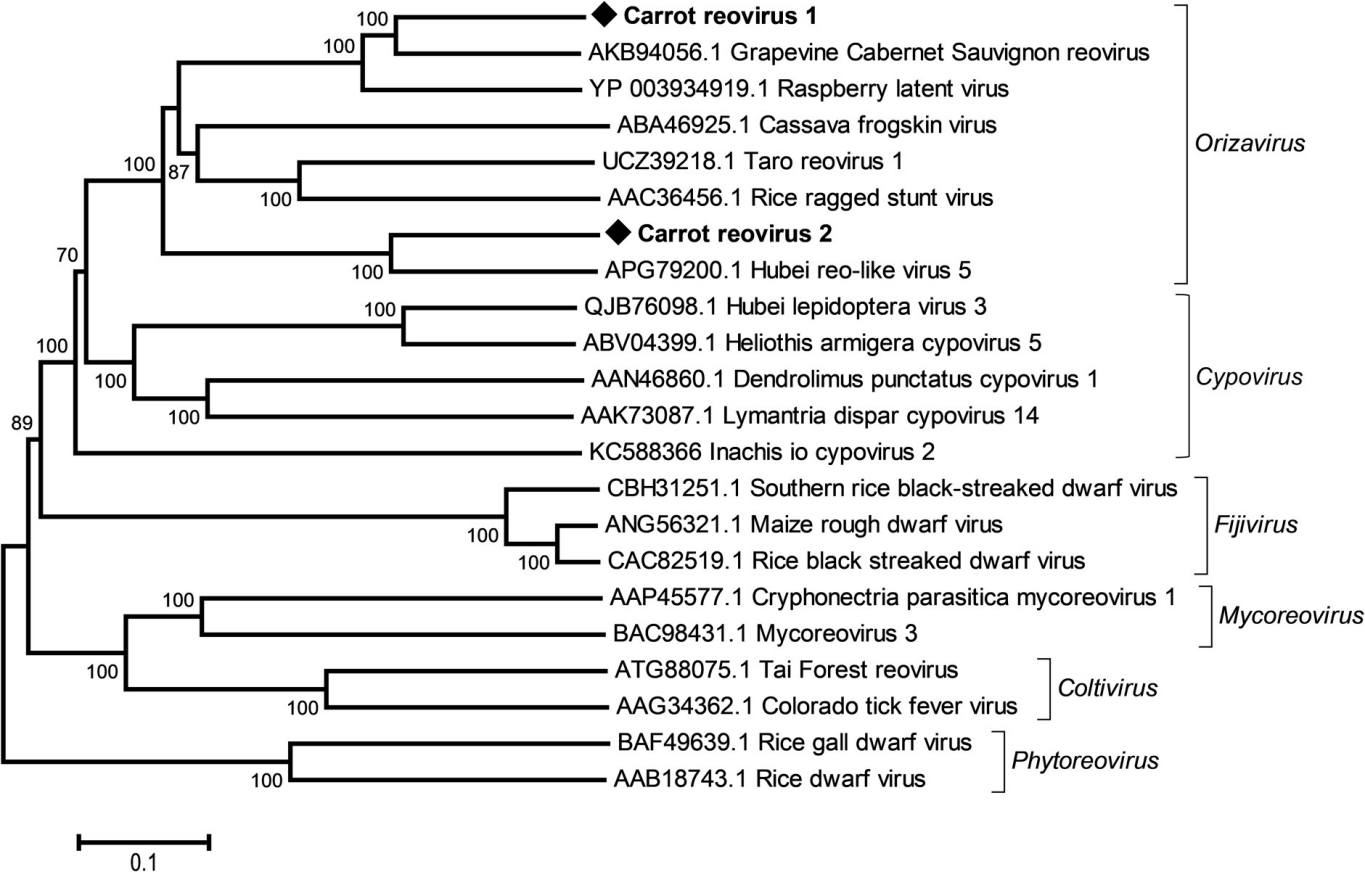
Neighbour-joining tree reconstructed from amino acid alignment of the RNA-dependent RNA polymerase of the newly identified carrot reovirus 1 and carrot reovirus 2 and of representative *Spinareoviridae* members and related unassigned viruses retrieved from GenBank. Bootstrap values above 70% (1000 replicates) are shown. The scale bar represents 10% amino acid divergence between sequences. Novel viruses identified in this study are indicated by black diamonds and accession numbers are shown for each reference sequence. Genera are shown on the right of lateral brackets.

Two novel viruses, carrot reovirus 1 (CaRV1) and carrot reovirus 2 (CaRV2), were identified in French wild carrot populations. Contigs for ten RNA genomic segments could be obtained for CaRV1, which was identified in three wild populations (FR20-18, FR20-20, FR20-21, S1 Table in S1 File) sampled in 2020 in France. On the other hand, only three genomic segments, including one encoding the viral RdRp, could be identified by Blast searches for CaRV2, which was identified in only two populations sampled in different parts of the north of France (populations FR20-19 and FR20-22, S1 and S5 Tables in S1 File). Both viruses show homologies to members of the family *Spinareoviridae* (previously *Reoviridae*) and group in separate subclusters of a clade composed of rice ragged stunt virus (RRSV, *Oryzavirus*) and unclassified plant-associated viruses, including grapevine Cabernet Sauvignon reovirus (GCSV) and raspberry latent virus (RpLV) (Fig 9). The latter was proposed to belong to a novel genus in the previously listed *Reoviridae* family [42]. Eight out of 10 obtained CaRV1 genomic segments appear to be full length and show nearly identical conserved ends as seen for RpLV [42], with the conserved tetranucleotide AGUU at the 5’ termini and the hexanucleotide GAAUAC at the 3’ termini. Pairwise RdRp aa identities of respectively 71% and 66% are obtained between CaRV1 and GCSV or RpLV, distances that are well below the 86–94% aa identities registered between maize rough dwarf virus, southern rice black-streaked dwarf virus and rice black streaked dwarf virus, which are all ICTV accepted species (S9 Table in S1 File). If a new genus is created at some point to include RpLV, CaRV1 would clearly be a candidate to be integrated in this genus, together with GCSV (Fig 9). In the case of the partial CaRV2 RdRp, the highest aa identity recorded, 68%, was with Hubei reo-like virus 5 (KX884720), a virus identified from insect metagenomic data.

### Other double-stranded RNA viruses identified in the carrot virome

**Fig 10:**
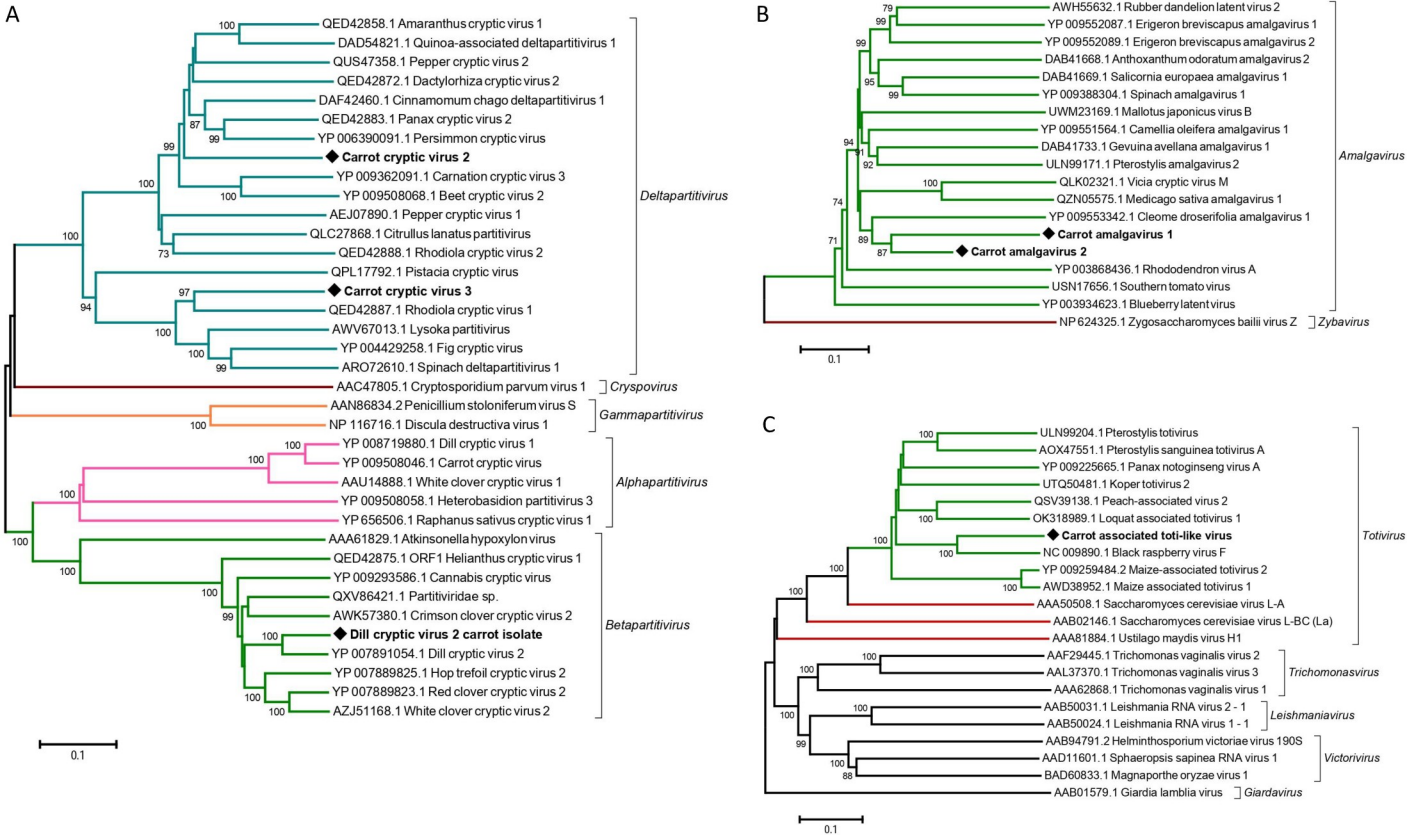
Neighbour-joining trees reconstructed from amino acid alignments of RNA-dependent RNA polymerase sequences of *Partitiviridae* members (A), *Amalgaviridae* members (B), and *Totiviridae* members (C). Bootstrap values above 70% (1,000x replicates) are shown. Scale bars represent 10% amino acid divergence between sequences. Colour coding denotes the different genera within a family or, for totiviruses, the host (green for plant-associated viruses, brown for fungi-associated viruses). Novel or tentatively novel viruses identified in this study are indicated by black diamonds and accession numbers are shown for each reference sequence. Genera are shown on the right of lateral brackets.

A few viruses of the family *Partitiviridae* have been reported to infect carrots. A member of the genus *Alphapartitivirus*, carrot cryptic virus (CaCV), has been reported in Germany [43] and unassigned *Partitiviridae* members have been reported in Japan [44], referred to as carrot temperate viruses 1–4 (CteV1-4). Unfortunately, no reference isolates or sequence information are available for the latter. In the 9^th^ Report of Virus Taxonomy [45], CteV1, CteV2 and CteV4 have been listed as members of the *Alphapartitivirus* genus and CteV3 in the genus *Betapartitivirus*. CaCV has been detected in a high proportion of cultivated (11/15, 73.3%) and off-type populations (6/7, 86%) but was only detected in one wild population sampled near Bordeaux (FR20-15, S1 Table in S1 File). Three other viruses from different *Partitiviridae* genera were also identified. A divergent variant of dill cryptic virus 2 (DiCV2) was detected in all three population types, infecting a higher proportion of wild carrot populations (6/23, 39%) than cultivated (2/15, 8%) or off type ones (1/7, 14%). It was geographically limited to the region Nouvelle-Aquitaine (S5 Table in S1 File). The DiCV2 carrot isolate here identified shows significant divergence from the type DiCV2 in GenBank and that was isolated from dill (NC_021147 and NC_021148), showing with it only 88% and 86% pairwise aa identity in the RdRp and CP, respectively (S10 Table in S1 File), which are borderline values for species demarcation according to the ICTV guidelines for the genus *Betapartitivirus* [45]. Phylogenetically, DiCV2 isolates cluster together with other betapartitiviruses and are clearly separated from other genera in the *Partitiviridae* family (Fig 10A). Two novel members of the *Deltapartitivirus* genus have also been identified in the carrot virome, representing to our knowledge the first report of deltapartitiviruses in carrot. These two novel viruses are referred to as carrot cryptic virus 2 (CaCV2) and carrot cryptic virus 3 (CaCV3) and typically possess two RNA segments of about 1.5 kb each. RNA1 codes for the viral RdRp and RNA2 for the CP. CaCV2 was detected in 8/23 (34.8%) cultivated and 4/15 wild populations (26.7%), both in the north and southwest of France (S5 Table in S1 File). It was also detected in one cultivated Spanish population (ES21-1A). The two contigs assembled for the genomic RNAs of CaCV2 have an average coverage of 31,529x (Table 1). CaCV2 is most closely related to persimmon cryptic virus (GenBank HE805113-14) and, based on the RdRp, clusters with other members of the *Deltapartitivirus* genus (Fig 10A). A similar clustering was observed when using the CP. The second novel deltapartitivirus CaCV3 was identified from a single wild carrot population in Spain (ES21-4, S1 Table in S1 File). Similar to CaCV2, the genomic contigs assembled had very deep average coverage (13,634x, Table 1). CaCV3 shows clear phylogenetic affinities to members of the genus *Deltapartitivirus* (Fig 10A). Highest pairwise RdRp aa identity (68%) was observed with persimmon cryptic virus (YP_006390090), while the corresponding value for the less conserved CP was only of 42% with rhodiola cryptic virus 1 (QED42889) (S10 Table in S1 File). However, these two novel viruses cluster in different subgroups within the *Deltapartitivirus* genus and their RdRp and CP proteins are very divergent (Fig 10A and S10 Table in S1 File). Given the very limited information available on CteV1-4, it is not possible to ascertain whether they might be similar to CaCV2 and CaCV3 reported here.

Contigs showing similarities to members of the genus *Amalgavirus* (family *Amalgaviridae*) were detected in several French wild and off-type populations in both sampling years. This virus was not identified from French cultivated populations or from Spanish carrots. A nearly complete genome of this new amalgavirus, referred to as carrot amalgavirus 1 (CaAV1), was obtained from a wild carrot population sampled in 2020 in France (FR20-15, S1 Table in S1 File). The obtained 3.2 kb contig (average coverage 929x, Table 1) harbors the two ORFs characteristic of amalgaviruses. ORF1 encodes a 44 kDa protein (398 aa) of unknown function and ORF2, translated in the +1 frame with respect to ORF1, codes for a 118 kDa (1066 aa) fusion protein containing the RdRp. The overlapping ORF1-ORF2 sequence of CaAV1 contains the +1 ribosomal frameshift motif conserved among amalgaviruses (UUU CGN) [46]. CaAV1 does not seem to be closely related to any previously reported amalgavirus and shows highest RdRp pairwise aa identity of 48% with Gevuina avellana amalgavirus 1 (DAB41733, S11 Table in S1 File).

A short contig of 777 nt (19x average coverage, Table 1) showing sequence similarity to members of the genus *Amalgavirus*, was assembled from the reads from the wild population sampled in Spain (ES21-4, S1 Table in S1 File). The sequence covers a central region of amalgavirus ORF2 and is most closely related to CaAV1 with 69% aa identity (S11 Table in S1 File), which is in the range for species demarcation (65–70% aa identity in the RdRp) for the *Amalgavirus* genus. This contig is therefore considered as tentatively representing a partial genome of a distinct amalgavirus, with the proposed name carrot amalgavirus 2 (CaAV2). Even though the sequence of CaAV2 is only partial, raising uncertainties about the divergence level when analyzing complete proteins, pairwise identity levels between recognized amalgavirus species in the region encompassed by the CaAV2 contig are similar to the CaAV1-CaAV2 divergence level, providing evidence that the two viruses likely correspond to different species. Both viruses cluster unambiguously within the *Amalgavirus* genus and away from Zygosaccharomyces bailii virus Z (GenBank NP_624325), the only member of the second genus recognized in the *Amalgaviridae* family (genus *Zybavirus*) (Fig 10B).

Two overlapping contigs of 923 nt and 4,254 nt were assembled from the reads from an off-type carrot population sampled in 2020 in Gironde (FR20-4, S1 Table in S1 File). The contigs were scaffolded into a near complete genome of 5,128 nt (8876x average coverage, Table 1). This contig harbors two ORFs: ORF1 encodes a 793 aa protein corresponding to the CP, and ORF2, which overlaps ORF1 by only one nucleotide, encodes a 69 kDa (855aa) protein with a RdRp conserved motif. This genomic organization is similar to that of viruses of the *Totiviridae* family, and the encoded proteins show affinities to those of *Toviviridae* members, so that it is proposed to refer to the identified virus as carrot-associated toti-like virus (CaaTLV). In a BLASTN search, CaaTLV shares highest identity with black raspberry F virus (BRVF, GenBank EU082131). Pairwise comparisons of its CP and RdRp confirm BRVF as the most closely related virus with 65% and 73% aa identity in the CP and RdRp, respectively (S12 Table in S1 File) and phylogenetic analyses demonstrate its affinities with a group of plant-associated totiviruses (Fig 10C). Although the pairwise protein identity levels are above the accepted molecular demarcation criteria (50% aa identity threshold), the viruses in question originate from different host species, which is a biological ICTV demarcation criterion. In addition, RdRp identity levels between accepted plant-associated totiviruses are often well above the 50% threshold (e.g. between peach-associated virus 2 (QSV39138) and loquat-associated totivirus 1 (OK318989) with 71% aa identity, S12 Table in S1 File), all providing evidence that CaaTLV may constitute a new species within the genus *Totivirus*.

### Unclassified putative novel *Riboviria* in the carrot virome

**Fig 11:**
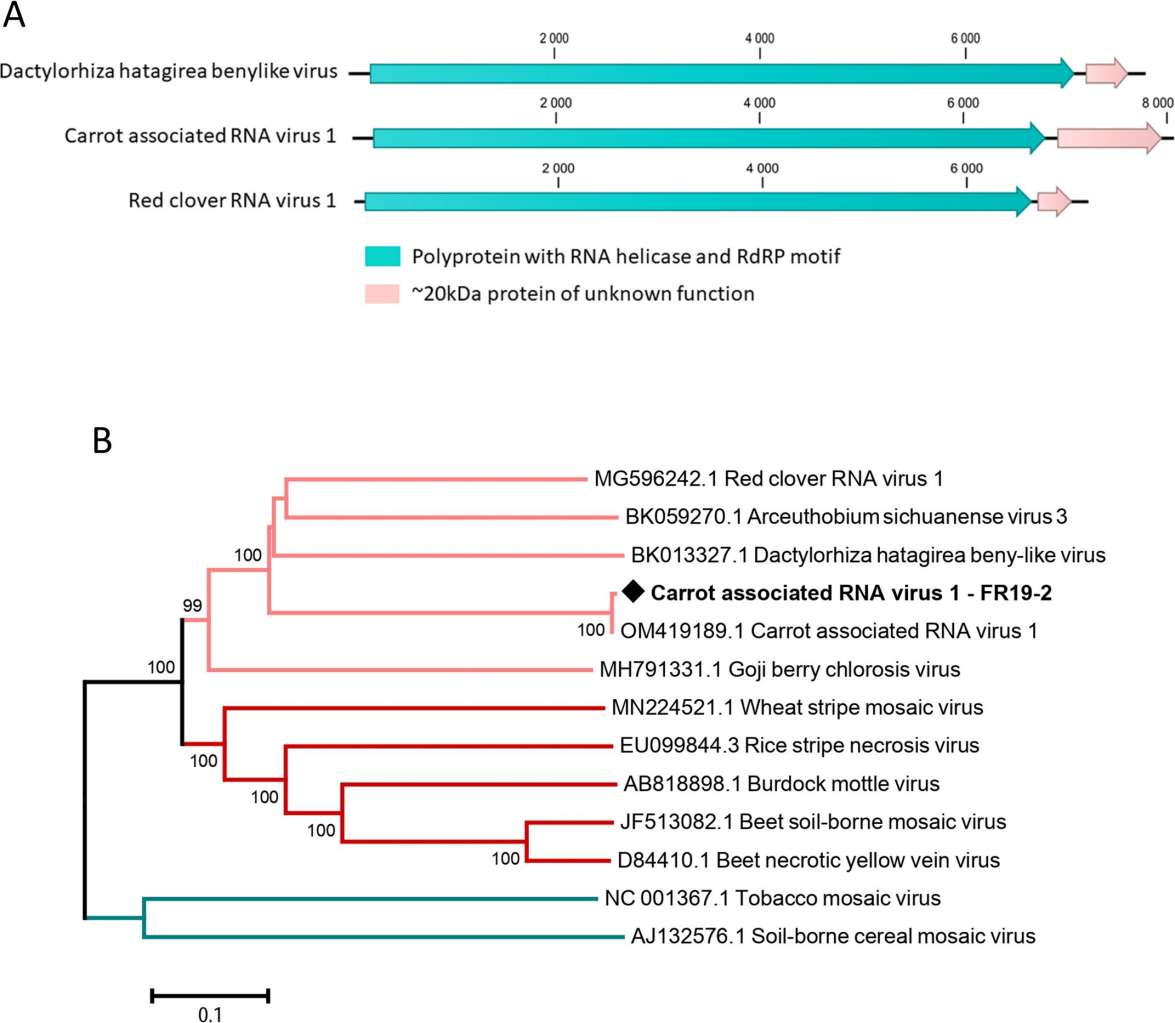
Genomic organization of unclassified novel viruses with homology to the *Benyviridae* family, including carrot-associated RNA virus 1 (A) and neighbour-joining tree reconstructed from amino acid alignment of the RNA-dependent RNA polymerase of carrot-associated RNA virus 1 and of closely related sequences from GenBank (clade indicated in light pink), of selected *Benyviridae* members (clade indicated in red) and of selected *Virgaviridae* members (clade indicated in blue) (B). Carrot-associated RNA virus 1 is indicated by a black diamond, accession numbers are given for each reference sequence and bootstrap values above 70% (1,000 replicates) are shown. The scale bar represents 10% amino acid divergence between sequences.

Several large ca. 8 kb nearly identical (ca. 99–100% nt identity) contigs were obtained from different French cultivated and off-type carrot populations in France. These contigs span the nearly complete genome of a novel virus showing homologies to members of the *Benyviridae* family. The selected reference contig is 8,068 nt long (average coverage 2927x, Table 1) and harbors two predicted ORFs, the largest encoding a 244 kDa protein (2,201 aa) with viral RNA helicase (PF01443) and RdRp (PF00978) motifs and the smallest, 3’ located, encoding a 18.5 kDa protein (165 aa) of unknown function (Fig 11A). The genome 3’end is polyadenylated, but the length of the polyA sequence was not determined. This genome organization is similar to that several other unclassified viruses in GenBank with homology to *Benyviridae* members (Fig 11B). In a BLASTN search, two recently released GenBank 1.2–1.3kb contigs (OM419188 and OM419189), referred to as carrot-associated RNA virus 1 (CaRNAV1) and obtained from a historical carrot sample collected in 2005 in Australia, show 99% and 100% identity to the sequence of the virus identified here, indicating that it should be referred to as carrot-associated RNA virus 1. The next most closely related viruses are red clover virus 1 (GenBank MG596242) and Dactylorhiza hatagirea beny-like virus (BK013327), which show pairwise RdRP aa identity levels of 43% and 39%, respectively (S13 Table in S1 File). Accordingly, CaRNAV1 groups together red clover RNA virus 1 and other unclassified viruses in a separate cluster from accepted *Benyviridae* members and shows more distant relationships to other viruses with rod-shaped virions of the *Virgaviridae* family (Fig 11B). The virus was very frequently detected in the collected field carrot populations in both countries, infecting 100% of cultivated and off-type populations in 2019 and 82% of cultivated and 100% of off type populations in 2020 in France as well as all four cultivated Spanish carrot populations.

Further identified putative novel Riboviria comprise carrot flavi-like virus 1 (CtFLV1) and carrot Ker-like virus (CaKLV). The molecular characterization of CtFLV1 and its geographical distribution has been recently described [47] while CaKLV, which shows homologies to viruses first identified in a viral metagenome from the Kerguelen island and likely belongs to a potential higher order viral taxon will be presented elsewhere (TC, AM, DS et al., manuscript in preparation).

## Discussion

In the past decade, several studies have begun to explore the largely underestimated viral diversity in unmanaged ecosystems and the role of wild plants as reservoirs of virus diversity [17–25]. The carrot pathosystem, consisting of cultivated and wild carrot populations, is particularly interesting with respect to metagenomic comparisons of virus communities due to the low genetic barrier to virus flow since both carrot types belong to the same plant species. This situation is further enriched by off-type carrots, that most likely represent hybrids between cultivated and wild carrots contaminating commercial carrot seed lots [48]. In a large-scale effort, we characterized the virome of 15 cultivated, 23 wild and seven off-type carrot populations from five different regions of France over two years. In addition, five cultivated and one wild carrot populations were sampled in Spain. Using a highly purified dsRNA-based HTS approach, we identified a very rich virome including 45 viruses of which 25 are novel or tentatively novel (Fig 1C). In the majority of cases, very long contigs, often representing near full-length genomes could be assembled from the reads derived from the pools of plants used, thanks to the deep average genome coverages achieved. With four exceptions, a coverage in excess of 150x was achieved for all novel viruses reported here (Table 1). This depth of coverage and ability to assemble very long contigs parallel results obtained in other virome studies [22, 25].

In a limited screening of public carrot RNASeq transcriptomic data in GenBank sequence reads archives (SRAs), a total of seven viruses were identified, five of which are in common with the present study, with the other two being the potyvirus carrot thin leaf virus and a novel *Partitiviridae* member distinct from those reported here. The range of carrot-infecting viruses with dsRNA genomes was extended through the identification of novel members of the families *Amalgaviridae*, *Totiviridae*, *Spinareoviridae* as well as two novel viruses of the *Deltaparitivirus* genus and one new host record for a divergent isolate of the betapartitivirus DiCV2. Most of the identified novel viruses are +ssRNA viruses that are aphid transmitted (Fig 1C), including members of the *Closteroviridae*, *Solemoviridae* and *Tombusviridae* families, as well as their associated subviral agents that are known to be involved in complex systems of mutual complementation and assistance. Umbraviruses (family *Tombusviridae*), that lack a CP gene, do not form virus particles and therefore rely on helper viruses, characteristically from the family *Solemoviridae* (formerly *Luteoviridae*) for encapsidation and aphid transmission [49]. The carrot motley dwarf disease complex is based on such an interaction between a polerovirus, CtRLV, and umbraviruses (CMoV or CMoMV), often in further association with an associated RNA (CtRLVaRNA). In this study, we identified a very diverse and rich spectrum of poleroviruses, umbraviruses and associated subviral agents. A total of four different poleroviruses was identified, two known (CtRLV, WtRLV) and two novel (CaPV1 and CaPV2), plus a new enamovirus (CaEV1). In addition, six different umbraviruses, three known (CMoV, CMoMV, WCMoV) and three new or potentially new (CaUV2 and CaUV3, CaUV1) were detected in different carrot populations in France, often in complex co-infections, when single plants were analyzed. The carrot virome was equally rich in subviral agents, with four different tombus-like-associated RNAs identified in French and Spanish carrot populations [38]. In addition, both linear and small circular, hammerhead-containing satellite RNAs [39] were detected. Synergistic interaction between poleroviruses and umbraviruses have been reported with destructive effects on different crops [50, 51] but there is little information on their precise interactions and even less so on the level of specificity or promiscuousness involved. Recent studies investigating disease complexes under field condition parallel our findings of novel polero- and umbraviruses in coinfections with known viruses involved in helper-dependent disease complexes such as the CMD complex. This is for example the case for a novel umbravirus and a novel satellite in mixed infections with the polerovirus potato leafroll virus (PLRV) causing the Tobacco Bushy Top Disease (TBTD) in Ethiopia [52]. Moveover, two novel poleroviruses, tobacco polerovirus 1 and tobacco polerovirus 2, have been identified in symptomatic TBTD affected plants coinfected with other viruses such as tobacco bushy top virus (genus *Umbravirus*), tobacco distorting vein virus (genus *Polerovirus*) and their associated satellites in China’s Yunnan province [53]. To gain deeper insights into virus prevalence and co-infection patterns, individual carrot plants of one cultivated and one wild population sampled in France in 2019 (FR19-7 and FR19-9, S1 Table in S1 File) were individually tested by specific RT-PCR and analyzed by dsRNA-based HTS (DS, TC, AM et al., in preparation). The results confirmed that plants were frequently coinfected by different known and novel poleroviruses, umbraviruses and associated subviral agents, suggesting complex and flexible interactions. Indeed, while arracacha latent virus E (ALVE, genus *Enamovirus*) was not observed in French or Spanish carrot viromes, arracacha latent virus E-associated RNA (ALVEaRNA) was frequently identified, often in coinfection with CtRLV, which likely fulfils a helper virus role, thus indicating some degree of plasticity in the association between different helper and dependent viruses [38]. Co-infections likely explain that several tentative recombination events could be detected among members of the *Polerovirus* genus, including a statistically highly significant recombination in the CP-readthrough (CP-RT) protein involving WCtRLV, CtRLV and the novel CaPV1. The CP-RT domain of the polerovirus genome has been shown to be the most variable genome part, with a high frequency of sites subject to positive selection, and has been suggested to be a major player in mediating adaptation to host plants and to aphid vectors [54]. Indeed, WCtRLV, together with CaPV1, was found exclusively in wild carrot populations, which in turn may be indicative of host adaptation.

Two closteroviruses were previously known from carrot, CYLV and CtCV1. CYLV was first isolated from carrots showing leaf yellowing in Japan [55] and is semi-persistently transmitted by *Cavariella* spp. [40]. similar as beet yellows virus (BYV), CYLV has 10 ORFs but in comparison to other closteroviruses, the genome positions of the p6 and p30 ORFs are reversed. The second already known carrot-infecting closterovirus, CtCV1 was identified in the frame of study investigating internal root necrosis in UK carrot crops using HTS [16]. In comparison to CYLV, CtCV1 lacks the p30 gene (Fig 7). We have identified two additional, closely related closteroviruses, CtCV2 and CtCV3 with an original genomic organization characterized by an HSP70h ORF located directly downstream of ORF1b (Fig 7). As pointed out by [40], five genes downstream of the RdRp gene (p6, HSP70h, p60, CP and CPm) are highly conserved among members of the *Closteroviridae* family but their order is not. A similar gene reshuffling is seen for example in the genera *Ampelovirus* and *Crinivirus* with a reversed order of the CPd and CP ORFs [40].

Another group of +ssRNA viruses identified is this study involves the family *Betaflexiviridae*. The genus *Chordovirus* was recently accepted within the family *Betaflexiviridae* (https://ictv.global/taxonomy) and comprises carrot chordovirus 1 and 2 (CChV1 and CChV2), identified in the UK [16], lettuce chordovirus 1 identified in lettuce in France [56] and hogweed virus 4 (OK032418) recently characterized from a historical UK hogweed sample [57]. We identified two novel chordoviruses, CChV3 and CChV4 as well as a novel vitivirus, CaVV1. Many viruses of the *Betaflexiviridae* family have no known insect or fungal vector. However, carlaviruses are generally transmitted by aphid vectors, while some vitiviruses have been reported to be transmitted by mealybugs [58] and by the aphids *Ovatus crataegarius* and *Cavariella* spp. [59]. Multiple studies have suggested that presence of a closterovirus or ampelovirus is required for insect-mediated transmission of vitiviruses [59, 60, for a review see 61]. No infection by closteroviruses was however detected in the carrot population in which CaVV1 was identified and it will be therefore of much interest to further investigate the transmission mode of the *Betaflexiviridae* family members identified in carrot populations and their potential dependence on other viruses for transmission.

Previously unknown putative novel Riboviria have been found in different carrot populations including the recently described CtFLV1 [47], carrot-associated RNA virus 1 and the carrot Ker-like virus (TC, AM, DS et al., in preparation). Two ca. 1.2 kb contigs of carrot-associated RNA virus 1 have been identified in a 17 years old carrot sample from Australia [62] that are nearly identical to the near complete genomes assembled here for this virus, suggesting a very limited diversity of this virus in both space and time. CaRNAV1 was also identified by datamining of two public SRA datasets, SRR5829255 (cultivated carrot cv. Kurada sequenced in China) and SRR11243946 (cultivated carrot sequenced in Sweeden). A RT-PCR screening of seedlings germinated from carrot seeds from different origins has confirmed CaRNAV1 low diversity and shown it to be seed-transmissible in carrot (DS, TC, AM et al., in preparation).

We did not identify any negative-stranded RNA viruses in the studied carrot viromes. While such viruses have been observed in several large virome studies [20–21, 25] they are usually much less frequent than positive-sense RNA viruses are sometimes are not observed [17]. There are indications that negative-stranded viruses are less efficiently detected than positive-stranded ones when using the dsRNA-based virome analysis strategy employed here (Schönegger *et al*., submited). The failure to detect such negative-stranded RNA viruses might therefore mark either their absence/low prevalence in the studied carrot viromes or, possibly, a methodological bias.

Most of the identified novel viruses showed preferential associations with wild carrots, either being exclusively identified from wild populations or infecting only a small proportion of cultivated populations (Table 1). This suggests that wild carrots represent a reservoir of viral diversity from which novel viruses attacking carrot crops could potentially emerge. It also suggests that despite the close taxonomic relationship linking cultivated and wild carrots, host adaptation or other unforeseen barriers may limit the flow of viruses between plants belonging to these two subspecies. Using HTS we have obtained a comprehensive picture of the carrot virome at the agroecological interface in two countries, France and Spain, which proved to be very diverse and rich, especially in viruses involved in complex interactions of mutual interdependencies for aphid transmission. Our results show that the carrot pathosystem provides a particularly interesting playground for further investigations of the poorly studied interplay between coinfecting helper and dependent viruses and of the evolutionary processes leading to synergistic or antagonistic virus-vector-host relationships under natural field conditions.

## Supporting information

S1 File The following supplementary tables are included as separate sheets in a single Supplementary Tables Excel file. S1 Table. Information on sampled carrot populations in France and Spain. **S2 Table.** RT-PCR and RACE primer pairs targeting known and novel viruses in the carrot virome. **S3 Table.** RNA-dependent RNA polymerase (RdRp) pairwise amino acid identities for carrot umbraviruses 1, 2 and 3 and selected genus *Umbravirus* members. **S4 Table.** RdRp pairwise amino acid identities for carrot poleroviruses 1 and 2, carrot enamovirus 1 and selected family *Solemoviridae* members. **S5 Table.** Geographical distribution of the various viruses identified in the carrot virome based on merged mapping of HTS data obtained from carrot populations from different regions of France and in Spain. Green squares indicate presence, red squares indicate absence of the virus in the respective locations. Novel viruses are indicated in red. **S6 Table.** RdRp and capsid protein (CP) pairwise amino acid identities for carrot chordoviruses 3 and 4, carrot vitivirus 1 and selected family *Betaflexiviridae* members. **S7 Table.** RdRp, heat-shock protein 70 (hsp70) and CP pairwise amino acid identities for carrot closteroviruses 2 and 3 and selected family *Closteroviridae* members. **S8 Table.** Full genome pairwise nucleotide identities and RdRp pairwise amino acid identities for carrot endornavirus 1 and selected family *Endornaviridae* members. **S9 Table.** RdRp pairwise amino acid identities for carrot reoviruses 1 and 2 and selected family *Spinareoviridae* members. **S10 Table.** RdRp pairwise amino acid identities for carrot cryptic viruses 2 and 3, dill cryptic virus 2 carrot isolate and selected family *Partitiviridae* members. **S11 Table.** RdRp fusion protein pairwise amino acid identities for Carrot_amalgaviruses 1 and 2 and selected family *Amalgaviridae* members. **S12 Table.** RdRp and CP pairwise amino acid identities for Carrot_associated_toti-like_virus and selected family *Totiviridae* members. **S13 Table.** RdRp pairwise amino acid identities for Carrot_associated_RNA_virus_1 and selected family *Benyviridae* members.
(XLSX)

S1 Fig Identified recombination events involving carrot infecting poleroviruses. (PDF)
