# Supplementary material for: Carrot populations in France and Spain host a complex virome rich in previously uncharacterized viruses
Source: PLoS One. 2023 Aug 16;18(8):e0290108. doi: 10.1371/journal.pone.0290108 (PMC10431682; doi:10.1371/journal.pone.0290108)
Supplement: S1 Fig — (PDF) [file pone.0290108.s002.pdf]

Umbraviruses

RDP pairwise identity plot

Recombination event: Carrot umbravirus 1 (CaUV1)

Major parent: Pastinaca umbravirus 1 (PasUV1)

Minor parent: Carrot mottle virus (CMoV)

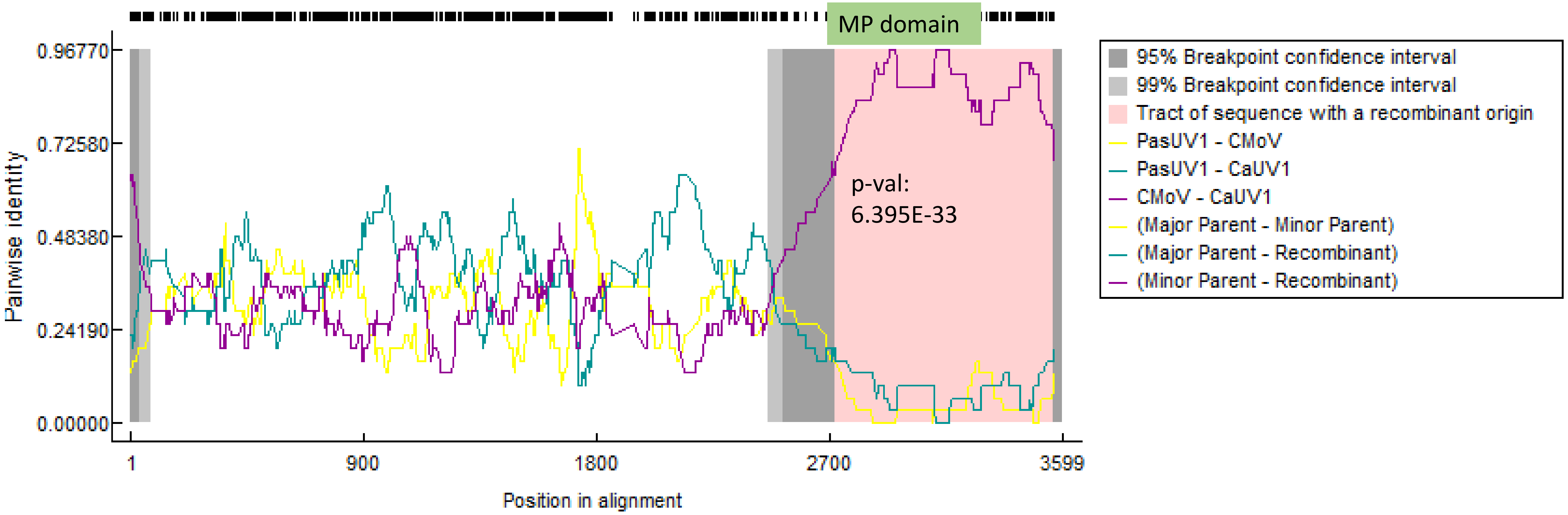

Region derived from **major parent:** 1-2717

Region derived from **minor parent**: 2718-3562

Poleroviruses

RDP pairwise identity plot

Recombination event: Wild carrot red leaf virus (WCtRLV)

Major parent: Carrot red leaf virus (CtRLV)

Minor parent: Carrot polerovirus 1 (CaPV1)

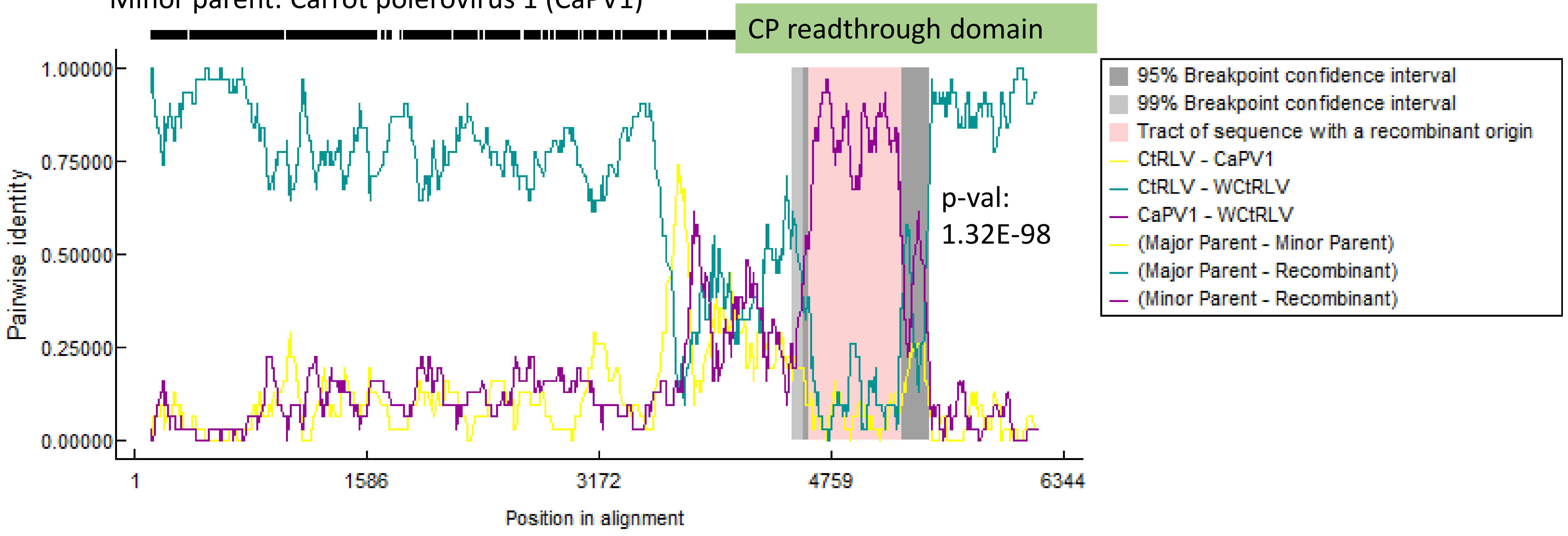

Region derived from **major parent:** 1-4601 and 5239-6344

Region derived from **minor parent**: 4602-5238

Recombination event: Trachispermum ammi polerovirus 1

Major parent: Carrot Polerovirus 1

Minor parent: Torrilis crimson leaf virus

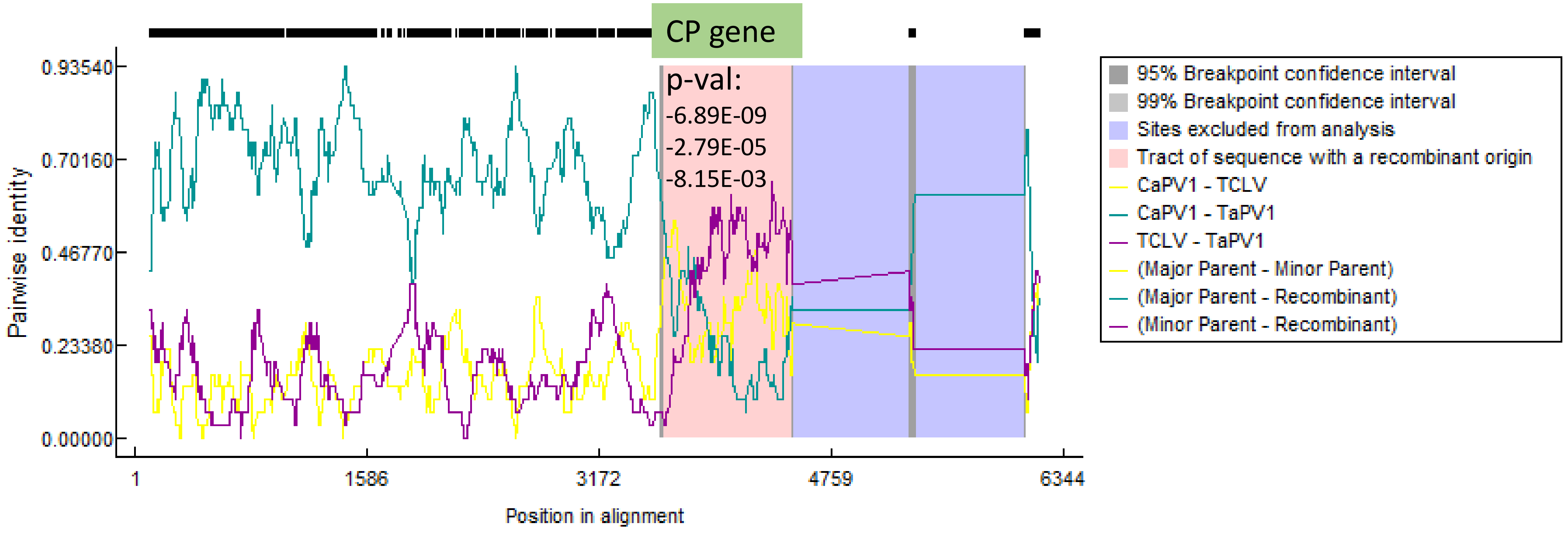

Region derived from **major parent:** 1-3617 and 4498-6344

Region derived from **minor parent**: 3618-4497
